# Network pharmacology of bioactives from *Sorghum bicolor* with targets related to diabetes mellitus

**DOI:** 10.1371/journal.pone.0240873

**Published:** 2020-12-31

**Authors:** Ki Kwang Oh, Md. Adnan, Dong Ha Cho

**Affiliations:** Department of Bio-Health Convergence, College of Biomedical Science, Kangwon National University, Chuncheon, Republic of Korea; Biotechnology HPC Software Applications Institute (BHSAI), UNITED STATES

## Abstract

**Background:**

*Sorghum bicolor* (SB) is rich in protective phytoconstituents with health benefits and regarded as a promising source of natural anti-diabetic substance. However, its comprehensive bioactive compound(s) and mechanism(s) against type-2 diabetes mellitus (T2DM) have not been exposed. Hence, we implemented network pharmacology to identify its key compounds and mechanism(s) against T2DM.

**Methods:**

Compounds in SB were explored through GC-MS and screened by Lipinski’s rule. Genes associated with the selected compounds or T2DM were extracted from public databases, and the overlapping genes between SB-compound related genes and T2DM target genes were identified using Venn diagram. Then, the networking between selected compounds and overlapping genes was constructed, visualized, and analyzed by RStudio. Finally, affinity between compounds and genes was evaluated via molecular docking.

**Results:**

GC-MS analysis of SB detected a total of 20 compounds which were accepted by the Lipinski’s rule. A total number of 16 compounds-related genes and T2DM-related genes (4,763) were identified, and 81 overlapping genes between them were selected. Gene set enrichment analysis exhibited that the mechanisms of SB against T2DM were associated with 12 signaling pathways, and the key mechanism might be to control blood glucose level by activating PPAR signaling pathway. Furthermore, the highest affinities were noted between four main compounds and six genes (FABP3-Propyleneglyco monoleate, FABP4-25-Oxo-27-norcholesterol, NR1H3-Campesterol, PPARA-β-sitosterol, PPARD-β-sitosterol, and PPARG-β-sitosterol).

**Conclusion:**

Our study overall suggests that the four key compounds detected in SB might ameliorate T2DM severity by activating the PPAR signaling pathway.

## 1. Introduction

Type 2 diabetes mellitus (T2DM) is a metabolic disease triggered by the complex interaction between genetic and/or environmental associations. Patients with T2DM have either insulin resistance (sugar transfer dysregulation into cells) or lack of optimal insulin secretion to sustain natural glucose levels [1]. T2DM can affect at all ages, 1 in 3 adults has prediabetes, causes serious physiological complications, and brings diabetes diagnosis economic difficulties [2–4]. The typical signs of T2DM patients include recurrent urination, increased thirst, exhaustion, increased appetite, and indistinct vision. The main etiology for T2DM is persistent hyperglycemia which provokes mitochondrial dysfunction that stimulates to abundant reactive oxygen species (ROS) formation in several tissues and pancreatic β cells [5, 6]. Such ROS accumulation in pancreatic β cells causes irreparable mitochondrial damage, resulting inhibition of insulin synthesis, and thus leading to diabetes progression by failing to produce enough level of insulin [7, 8]. However, the anti-diabetic drug pathways include stimulating the insulin synthesis, inhibiting the production of endogenous glucose, and blocking carbohydrate absorption from intestine [9, 10]. Currently, six classes of oral antidiabetic drugs, including Metformin, Glimepiride, Repaglinide, Pioglitazone, Sitagliptin, and Acarbose [11] are available which are reported to have serious side effects such as anorexia, nausea, dyspeptic episodes, and diarrhea [12, 13]. Hence, the quest for potential drugs has now become more concentrated. In this regard, a rich source of phytoconstituents with health benefits may be the prospective candidate for T2DM intervention.

For the past few years, a report has shown that wholegrains including sorghum (*Sorghum bicolor* (L.) Moench) are the potential alternatives to ameliorate T2DM symptoms [14]. *Sorghum bicolor* (SB) contains a number of secondary metabolites which are reported to be effective in preventing various metabolic diseases, such as cancers, T2DM, obesity, and hyperglycemia [15, 16]. A recent research demonstrated that oral treatment of SB (0.5% and 1%) noticeably reduced the low density lipoprotein cholesterol, triglycerides, and glucose level via PPARG in mice fed a high-fat diet, conversely, expression level of PPARG elevated to 1% [17]. Some reports expound that sorghum extracts at 1.0 mg/ml activates PPAR binding in mouse macrophage cell line; subsequently, PPAR agonists have been emerged as candidates against metabolic dysfunctions with T2DM [18, 19]. Lecka-Czernik B reported that Aleglitazar, being developed by Roche Holding, is a dual agonist for PPARD and PPARA for the promising multiple treatment of hyperglycemia and dyslipidemia with T2DM patients [20]. It is evident that a dual PPAR agonist treatment is more potent than a single agonist treatment. A human study on sub-health condition with prediabetes confirmed that SB administration significantly decreased the level of glucose by 35% [21]. Although literature survey revealed that diabetes and obesity complications can be controlled upon consumption of SB [22, 23]; however, its mechanism(s) against T2DM has not been explored to date. Therefore, the research on bioactive compound(s) and pathways of SB against T2DM should be justified in scientific testing, in order to maintain its pharmacological complements for T2DM.

Network pharmacology is a structured methodical mode, which can investigate the interactive networking elements such as compounds, genes, proteins, and diseases [24, 25]. Network pharmacology can decode the mechanism of compounds with a multifunctional point of view, which highlights the interaction of diverse factors, instead of “one target, one compound” [26]. The network pharmacology is therefore a valuable approach to identify potential lead compounds (from natural sources) with specific mechanism of action for the prevention of various disease, and mostly to elucidate the synergistic impact of bioactive compounds [27]. Zhang B. et al. reported that the rapid development of bioinformatics, systems biology, and poly-pharmacology contributes greatly to network-based drug discovery, which is regarded as a cost-effective drug development method [28]. A report explicates that the network pharmacology is used as a powerful tools to identify the mechanisms of actions between traditional Ge-Gen-Qin-Lian decoction (GGQLD) formula and target genes [29]. In our study, network pharmacology was used to evaluate the bioactive compounds and mechanism(s) of SB against T2DM. Firstly, GC-MS analysis of SB was conducted to identify the bioactive compounds and their “drug-likeness” property was screened by Lipinski’s rule [30]. Secondly, genes related to selected compounds or T2DM were identified using public databases, and overlapping genes between genes related to SB and T2DM target genes were also identified. Thirdly, genes related to a hub signaling were selected by analyzing gene set enrichment analysis. Finally, the selected genes were implemented for molecular docking analysis to find the most potent candidates of SB against T2DM.

## 2. Materials and methods

### 2.1 Plant preparation, extraction

The *Sorghum bicolor* (SB) was purchased from Chuncheon local market, Korea. The collected SB was dried and powdered using electric blender. Approximately 500 g of SB powder was soaked in 500 mL of 100% methanol (Daejung, Korea) for 3 days and repeated for 3 times to collect extraction. The solvent extract was collected, filtered, and evaporated using a vacuum evaporator (IKA- RV8, Japan). The evaporated sample was dried under a boiling water bath (IKA-HB10, Japan) at 40°C to obtain yield.

### 2.2 GC-MS analysis

Agilent 7890A was used to carry out GC-MS analysis. GC was equipped with a DB-5 (30m×0.25mm×0.25μm) capillary column. Initially, the instrument was maintained at a temperature of 100°C for 2.1 minutes. The temperature was risen to 300°C at the rate of 25°C/min and maintained for 20 minutes. Injection port temperature and helium flow rate were ensured as 250°C and 1.5 ml/min, respectively. The ionization voltage was 70 eV. The samples injected in split mode at 5:1. MS scan range was set at 35–550 (m/z). The fragmentation patterns of mass spectra were compared with those stored in the using W8N05ST Library MS database. The percentage of each compound was calculated from the relative peak area of each compound in the chromatogram. The concept of integration used the ChemStation integrater algorithms.

### 2.3 Chemical compounds database construction, drug-likeness, and oral bioavailability filtering

The information of chemical compounds from SB was identified by utilizing GC-MS analysis which were filtered according to the Lipinski’s rule through SwissADME (http://www.swissadme.ch/) to identify “Drug-likeness” property and oral bioavailability score. The PubChem (https://pubchem.ncbi.nlm.nih.gov/) was utilized for identification of the SMILES (Simplified Molecular Input Line Entry System) of compounds.

### 2.4 Target genes related to selected compounds or T2DM

Based on the SMILES, target genes linked to the compounds were selected through both Similarity Ensemble Approach (SEA) (http://sea.bkslab.org/) and Swiss Target Prediction (STP) (http://www.swisstargetprediction.ch/) with "*Homo Sapiens*” mode. T2DM related genes were identified by DisGeNET (https://www.disgenet.org/search) and OMIM (https://www.ncbi.nlm.nih.gov/omim) databases. The overlapping genes between compounds of SB and T2DM target genes were identified and visualized by VENNY 2.1 (https://bioinfogp.cnb.csic.es/tools/venny/).

### 2.5 Network construction of interacted overlapping genes

Through STRING (https://string-db.org/) analysis, the overlapping genes were closely correlated, and the signaling pathways of overlapping genes were analyzed by RStudio bubble chart. Networking between bioactive compounds and genes of SB against T2DM identified a hub signaling pathway.

### 2.6 Preparation for molecular docking of ligand molecules

The ligand molecules were converted.sdf from PubChem into.pdb format using Pymol, and the ligand molecules were converted into.pdbqt format through Autodock.

### 2.7 Preparation for molecular docking of target proteins

Six target proteins of T2DM i.e. FABP3 (PDB ID: 5HZ9), FABP4 (PDB ID: 3P6D), NR1H3 (PDB ID: 2ACL), PPARA (PDB ID: ASP6), PPARD (PDB ID: 5U3Q), PPARG (PDB ID: 3E00) were selected on STRING via RCSB PDB (https://www.rcsb.org/). The proteins selected as.PDB format converted into.pdbqt format via Autodock (http://autodock.scripps.edu/).

### 2.8 Ligand- protein docking

The ligand molecules were docked with target proteins utilizing autodock4 by setting-up 4 energy range and 8 exhaustiveness as default to obtain 10 different poses of ligand molecules [31]. The 2D binding interactions was identified through LigPlot+ v.2.2 (https://www.ebi.ac.uk/thornton-srv/software/LigPlus/). After docking, ligands of lowest binding energy were selected to visualize the ligand-protein interaction in Pymol.

## 3. Results

### 3.1 Potential bioactive compounds from SB

**Fig 1:**
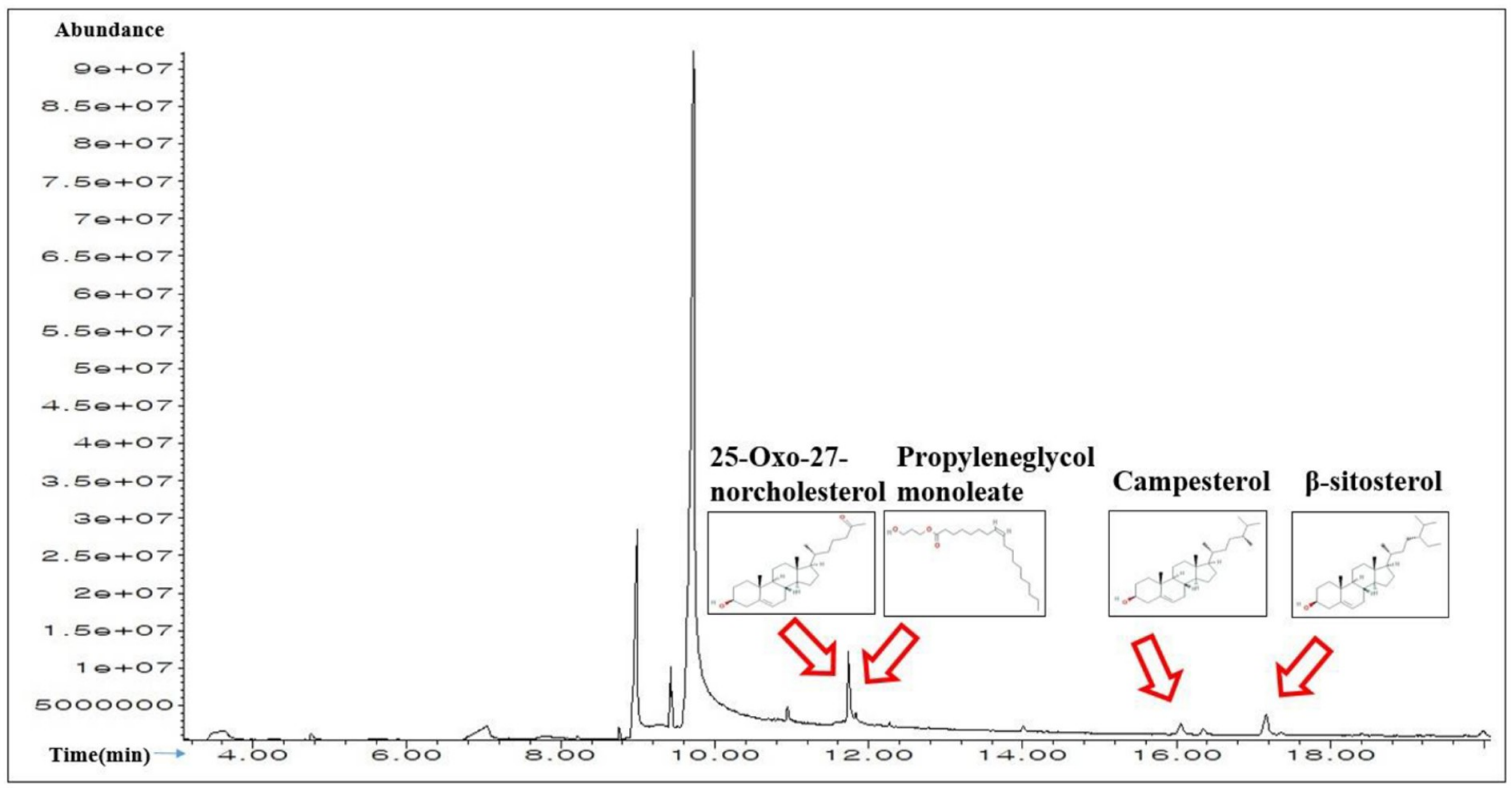
A typical GC-MS chromatogram of the chemical compounds of the methanol extract of SB with indication of 4 main chemical constituents.

A total of 20 compounds in SB were detected by the GC-MS analysis (Fig 1), and the name of compounds, retention time, peak area (%) are enlisted in Table 1. All 20 compounds were checked and accepted by Lipinski’s rule (Molecular Weight ≤ 500g/mol; Moriguchi octanol-water partition coefficient ≤4.15; Number of Nitrogen or Oxygen ≤10; Number of NH or OH ≤5), and all compounds have satisfactory “Abbott Bioavailability Score (> 0.1)” identified through SwissADME (Table 2).

**Table 1:** A list of the identified 20 chemical compounds from SB through GC-MS.

No.	Compound	Pubchem ID	Retention time	Area (%)
1	Glycerin	753	3.606	2.34
2	Glyceraldehyde	751	4.222	0.19
3	2,3-Dihydro-3,5-dihydroxy-6-methyl-4H-pyran-2-one	67452387	4.76	0.35
4	β-D-ribofuranosyl-cytosine	596	7.039	3.25
5	Butanoic acid, ethyl ester	7762	7.318	0.11
6	2-Propanol, 1-chloro-3-(1-methylethoxy)	94290	7.818, 7.847	0.84
7	Oxazole, 5-hexyl-2,4-dimethyl-	573438	8.222	0.15
8	Hexadecanoic acid, methyl ester	8181	8.76	0.24
9	Palmitic acid	985	8.991,9.270,9.308	13.67
10	Methyl linoleate	5284421	9.424	2.38
11	Linoleic acid	5280450	10.106,10.414,10.943	68.2
12	2-Stearoylglycerol	79075	10.943	0.72
13	Propyleneglycol monoleate	5365625	11.741	3.02
14	25-Oxo-27-norcholesterol	165617	11.837	0.95
15	Cholesterol	5997	12.279	0.14
16	5,8-Dimethyltocol	86052	14.01	0.26
17	Campesterol	173183	16.058	0.77
18	Stigmasterol	5280794	16.356	0.42
19	β-sitosterol	222284	17.164	1.52
20	4,5-Dihydroxycoumarin	54690194	19.991	0.28

**Table 2:** Physicochemical properties of the 20 compounds for good oral bioavailability.

No.	Compounds	Lipinski Rules				Lipinski’s Violations	Biavailability Score
		MW	HBA	HBD	MLog P		
		< 500	< 10	≤ 5	≤ 4.15	≤1	> 0.1
1	Glycerin	92.09	3	3	-1.51	0	0.55
2	Propanal, 2,3-dihydroxy-	90.08	3	2	-1.66	0	0.55
3	2,3-Dihydro-3,5-dihydroxy-6-methyl-4H-pyran-2-one	144.13	4	2	-0.96	0	0.55
4	β-D-ribofuranosyl-cytosine	243.22	6	5	-2.29	0	0.55
5	Butanoic acid, ethyl ester	116.16	2	0	1.27	0	0.55
6	2-Propanol, 1-chloro-3-(1-methylethoxy)	152.62	2	1	0.96	0	0.55
7	Oxazole, 5-hexyl-2,4-dimethyl-	181.27	2	0	1.92	0	0.55
8	Hexadecanoic acid, methyl ester	270.45	2	0	4.44	1	0.55
9	Palmitic acid	256.42	2	1	4.19	1	0.85
10	Methyl lineoleate	294.47	2	0	4.70	1	0.55
11	Linoleic acid	280.45	2	1	4.47	1	0.85
12	2-Stearoylglycerol	358.56	4	2	3.63	0	0.55
13	Propyleneglycol monoleate	340.54	3	1	4.37	1	0.55
14	25-Oxo-27-norcholesterol	386.61	2	1	5.1	1	0.55
15	Epicholesterol	386.65	1	1	6.34	1	0.55
16	5,8-Dimethyltocol	416.68	2	1	5.94	1	0.55
17	Campesterol	400.68	1	1	6.54	1	0.55
18	(E)-23-ethylcholesta-5,22-dien-3β-ol	412.69	1	1	6.62	1	0.55
19	β-sitosterol	414.71	1	1	6.73	1	0.55
20	4,5-Hydroxycoumarin	178.14	3	1	1.04	0	0.55

**MW**, Molecular Weight (g/mol); **HBA**, Hydrogen Bond Acceptor; **HBD**, Hydrogen Bond Donor; **LogP**, Lipophilicity; **Bioavailability Score**, the ability of a drug or other substance to be absorbed and used by the body.

### 3.2 Overlapping genes between SEA and STP linked to 20 compounds

**Fig 2:**
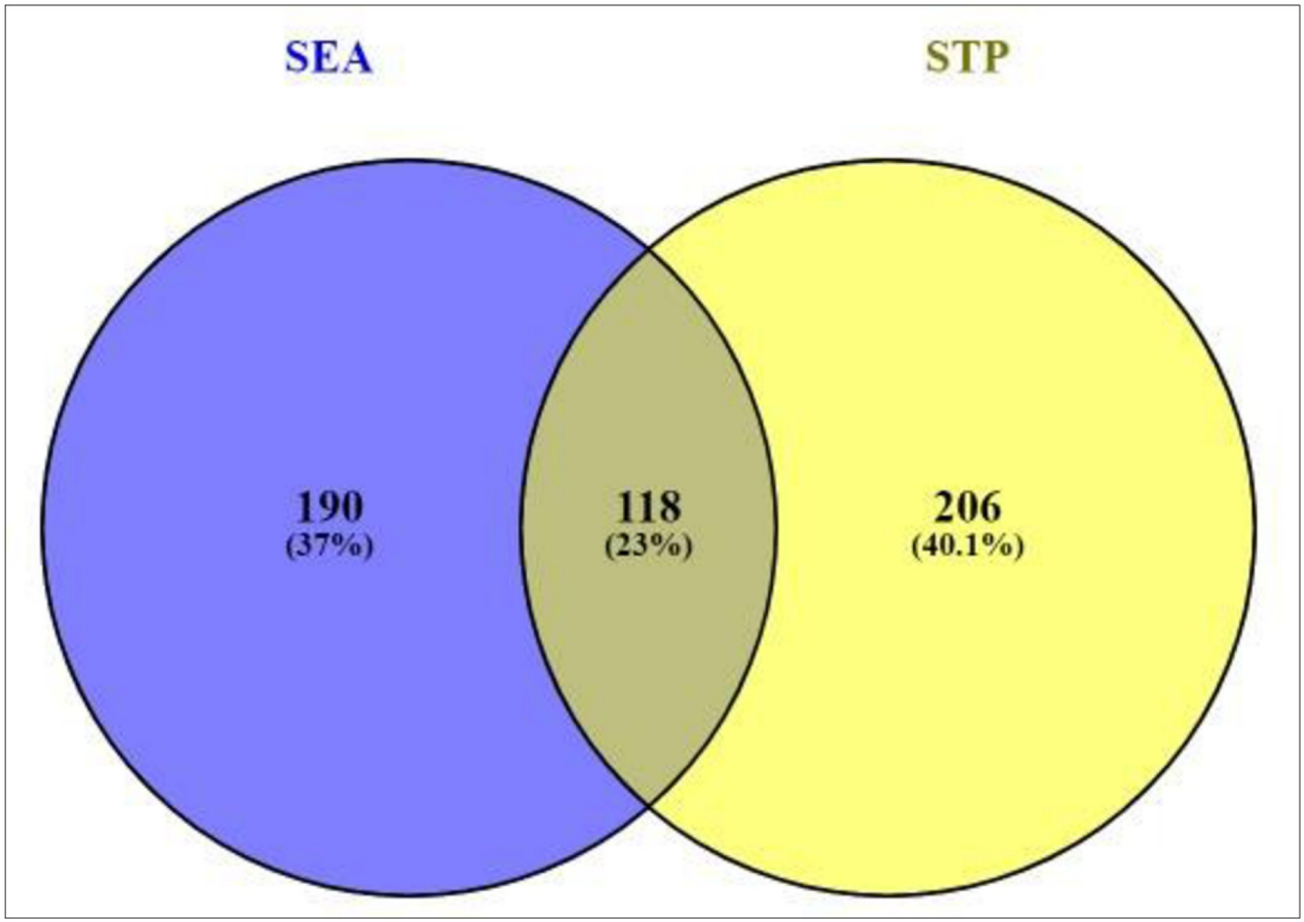
Overlapping genes (118genes) between SEA (308 genes) and STP (324 genes).

Based on the SMILES, a total of 308 genes from SEA and 324 genes from STP linked to 20 compounds were extracted (S1 Table). The result of Venn diagram exhibited that 118 genes were overlapped between the two public databases (Fig 2).

### 3.3 Overlapping genes between T2DM-related genes and the 118 overlapping genes

**Fig 3:**
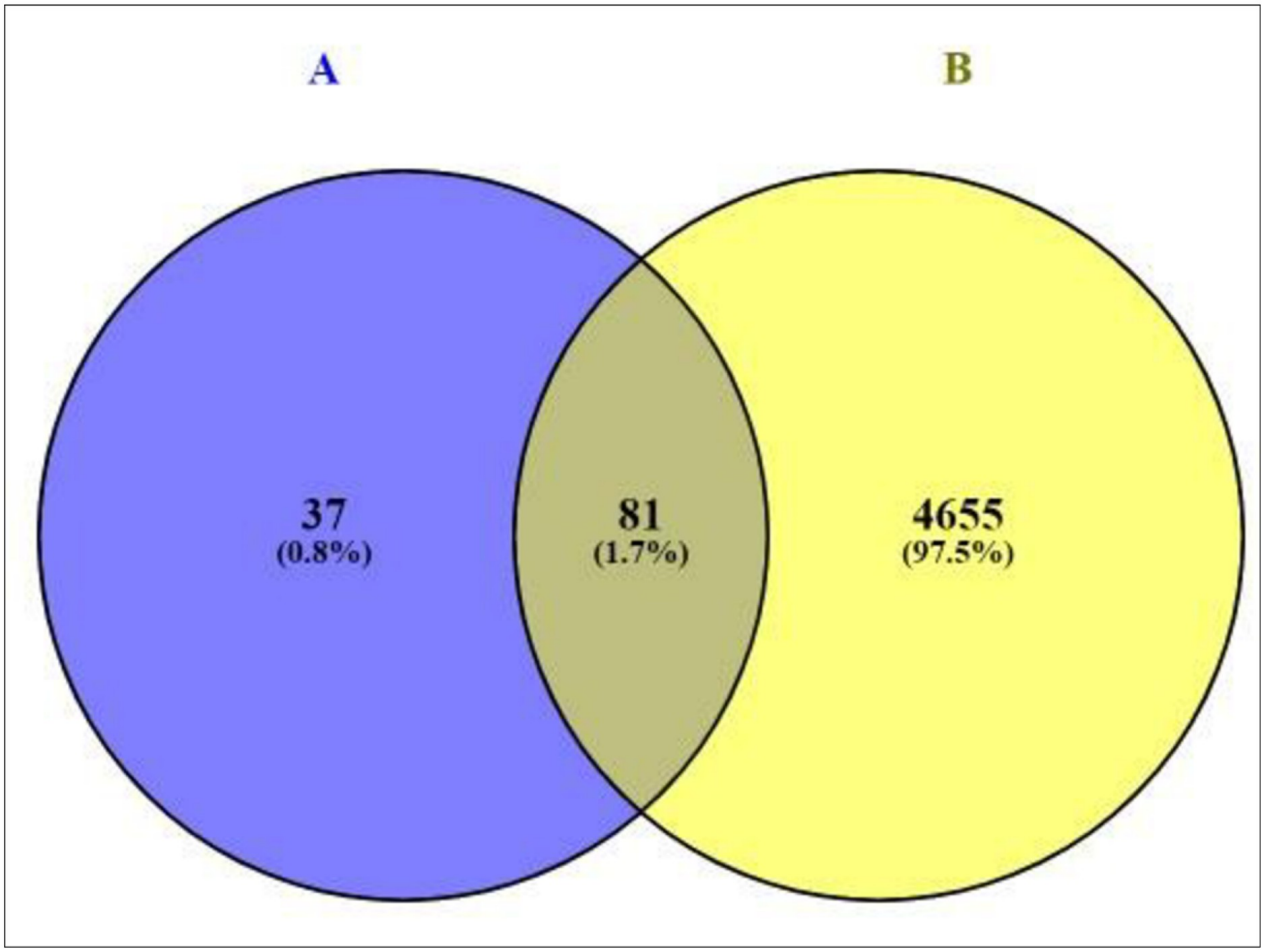
Overlapping genes between 118 overlapping genes (A) and T2DM related genes (4,736 genes).

A total of 4,736 genes related to T2DM were sorted by retrieving DisGeNET and OMIM databases (S2 Table). The result of Venn diagram unveiled that 81 overlapping genes was identified between 4,763 genes related to T2DM and the 118 overlapping genes (Fig 3), (S3 Table). As shown in S4 Table, a total of 81 overlapping genes linked to 16 compounds from aforementioned 20 compounds were identified, retrieving from both SEA and STP public databases, and no genes were found associated with other 4 compounds (Glycerin; Glyceraldehyde; 2,3-dihydro-3,5-dihydroxy-6-methyl-4H-pyran-2-one; and 2-propanol, 1-chloro-3-(1-methylethoxy)) in the two databases.

### 3.4 Gene-gene network analysis of 16 compounds of SB against T2DM

**Fig 4:**
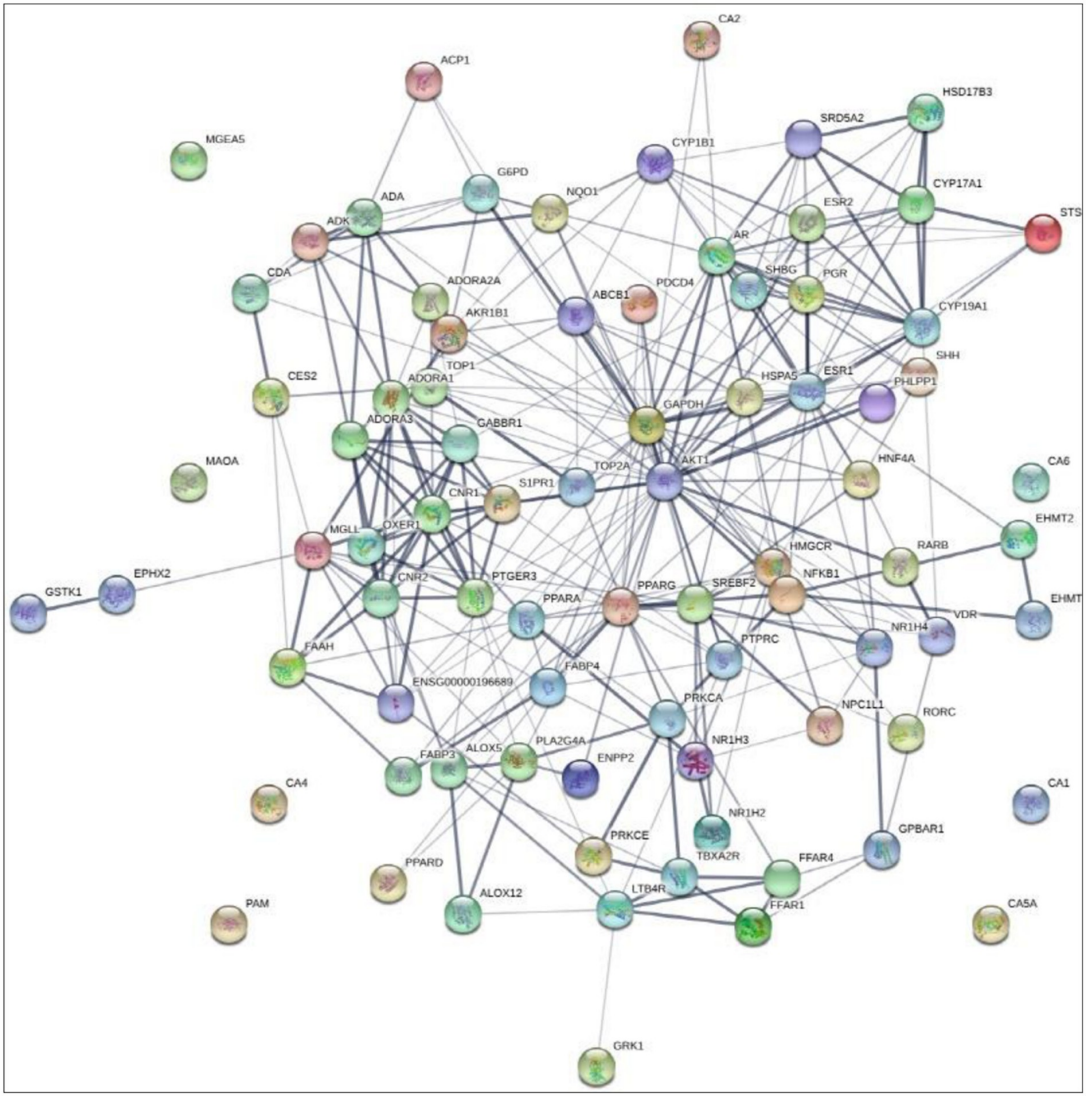
Gene-gene interaction of final overlapping 81 genes (81 nodes and 301edges) in SB against T2DM.

**Fig 5:**
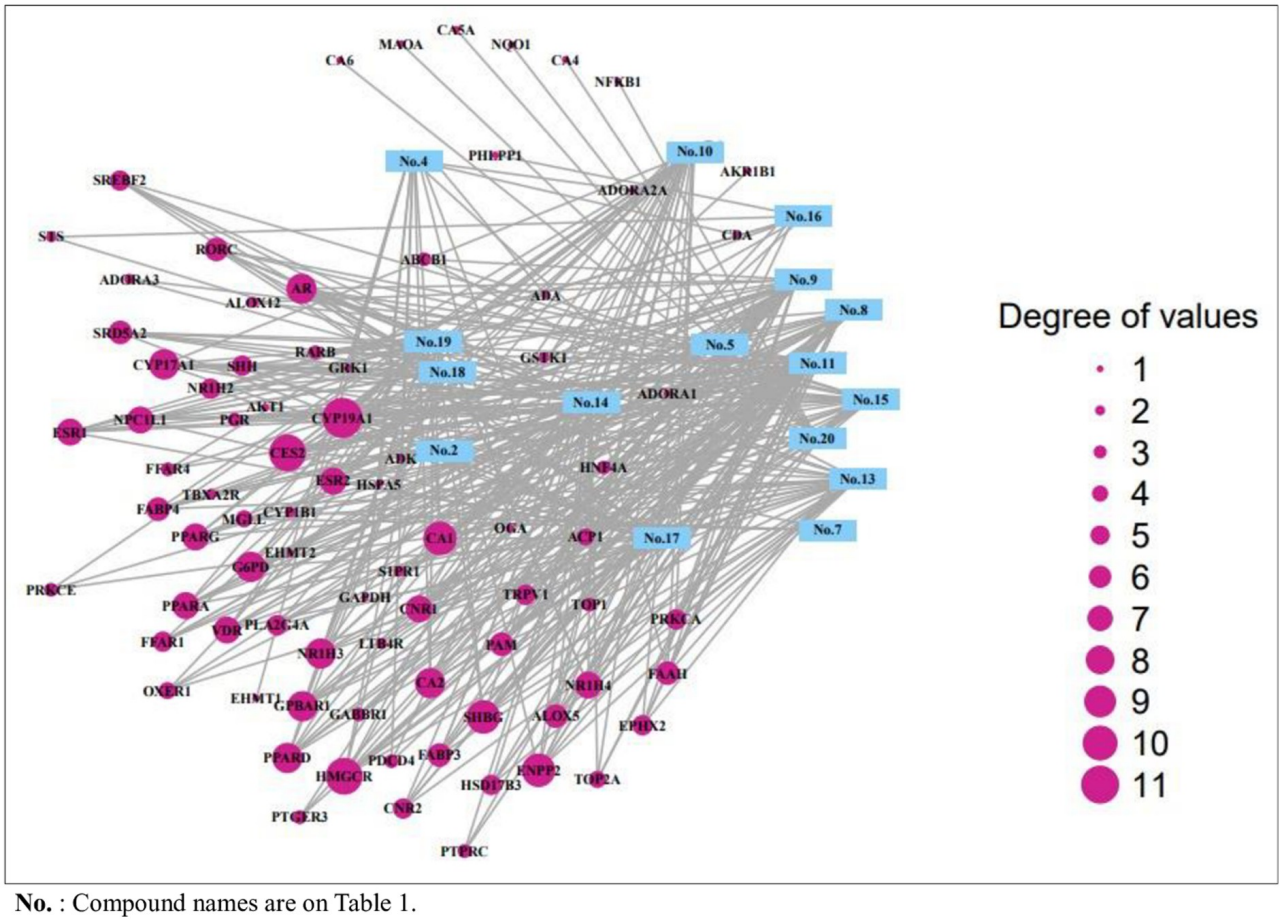
The interaction between 16 compounds and 81 genes (94 nodes and 356 edges) on T2DM.

The final overlapping 81 genes were closely linked to each other with 81 nodes and 347 edges (Fig 4). The interaction between 16 compounds and 81 genes resulted with 97 nodes and 347 edges (Fig 5), which indicated that the therapeutic efficacy of SB on T2DM. The 16 compounds were classified as five steroid derivatives (25-Oxo-27-norcholesterol, Cholesterol, Campesterol, (E)-23-ethylcholesta-5,22-dien-3β-ol, and β-sitosterol), four fatty acyls (Palmitic acid, Methyl lineoleate, Linoleic acid, and Propylengeglycol monoleate), two fatty acid esters (Butanoic acid-ethyl ester and Hexadecanoic acid-ethyl ester), one organooxygen (Propanal, 2,3-dihydroxy-), one prenol lipid (5,8-Dimethyltocol), one pyrimidine nucleoside (β-D-ribofuranosyl-cytosine), one azole (Oxazole, 5-hexyl-2,4-dimethyl), and one coumarin derivative (4,5-Dihydroxycoumarin).

### 3.5 Signaling pathways and finding of a hub signaling of SB against T2DM

**Fig 6:**
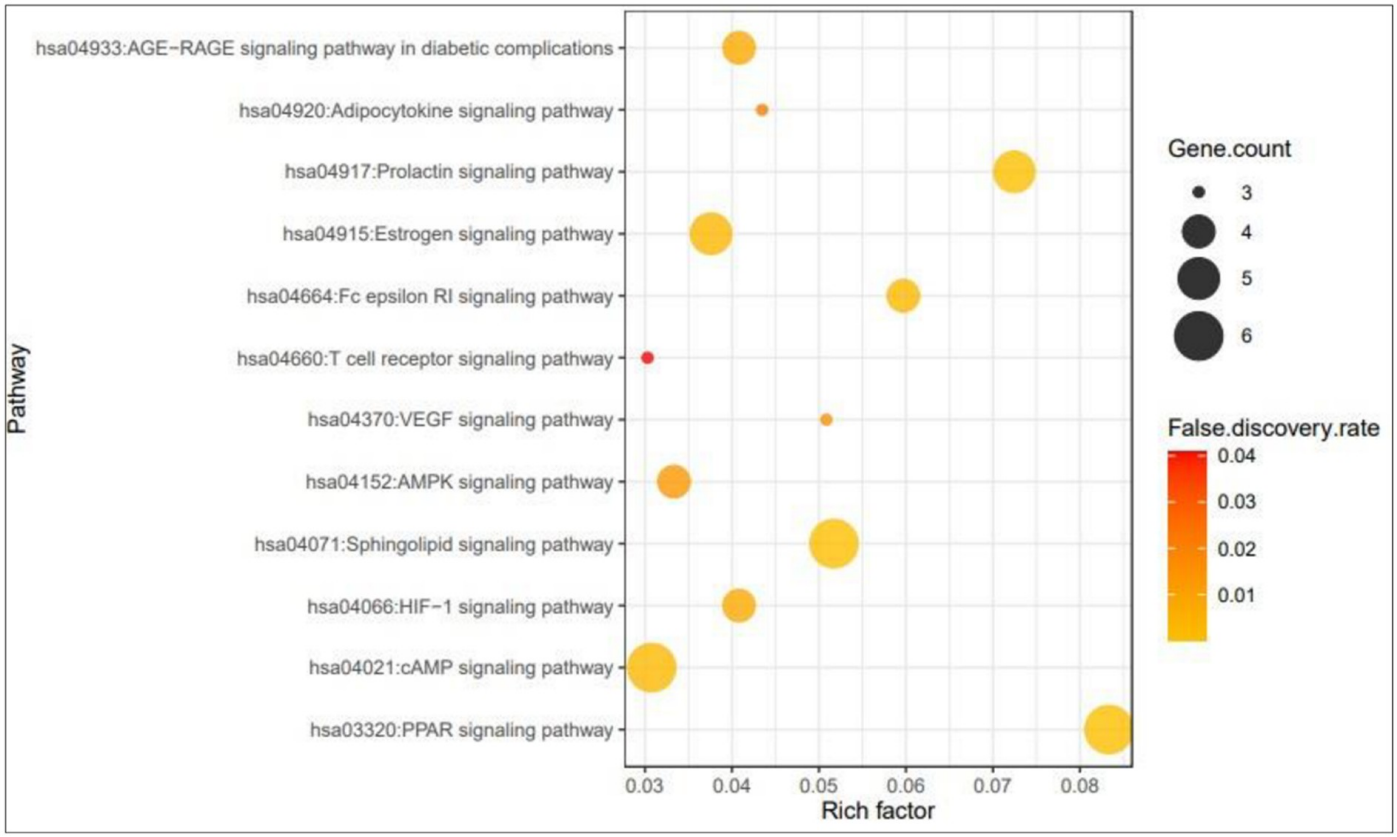
Bubble chart of 12 signaling pathways linked to the occurrence and progression of T2DM.

The result of KEGG pathway enrichment analysis demonstrated that 81 genes were related to 12 signaling pathways (False Discovery Rate < 0.05). The 12 signaling pathways were directly related to the progression of T2DM and indicated that these 12 signaling pathways might be the key pathways of SB against T2DM. The description of 12 signaling pathways is shown in Table 3. Additionally, a bubble chart suggested that PPAR signaling pathway might be a hub signaling pathway of SB against T2DM (Fig 6).

**Table 3:** Target genes in 12 signaling pathways enrichment related to T2DM.

KEGG ID	Target genes	False Discovery Rate
hsa04933:AGE-RAGE signaling pathway in diabetic complications	AKT1,NFKB1,PRKCA,PRKCE	0.0075
hsa04920:Adipocytokine signaling pathway	AKT1,PPARA,NFKB1	0.0208
hsa04917:Prolactin signaling pathway	ESR1,ESR2,AKT1,NFKB1,CYP17A1	0.00031
hsa04915:Estrogen signaling pathway	AKT1,GABBR1,ESR1,PGR,ESR2	0.0028
hsa04664:Fc epsilon RI signaling pathway	AKT1,ALOX5,PLA2G4A,PRKCA	0.0021
hsa04660:T cell receptor signaling pathway	AKT1,NFKB1,PTPRC	0.0408
hsa04370:VEGF signaling pathway	AKT1,PRKCA,PLA2G4A	0.0155
hsa04152:AMPK signaling pathway	AKT1,HNF4A,PPARG,HMGCR	0.013
hsa04071:Sphingolipid signaling pathway	ADORA1,S1PR1,AKT1,NFKB1,PRKCA,PRKCE	0.00029
hsa04021:cAMP signaling pathway	ADORA1,GABBR1,AKT1,PTGER3,PPARA,NFKB1	0.0022
hsa03320:PPAR signaling pathway	FABP3,FABP4,NR1H3,PPARA,PPARD,PPARG	0.0000419
hsa04066:HIF-1 signaling pathway	GAPDH,AKT1,NFKB1,PRKCA	0.0075

### 3.6 Molecular docking investigation of 6 genes and 4 compounds related to PPAR signaling pathway

**Fig 7:**
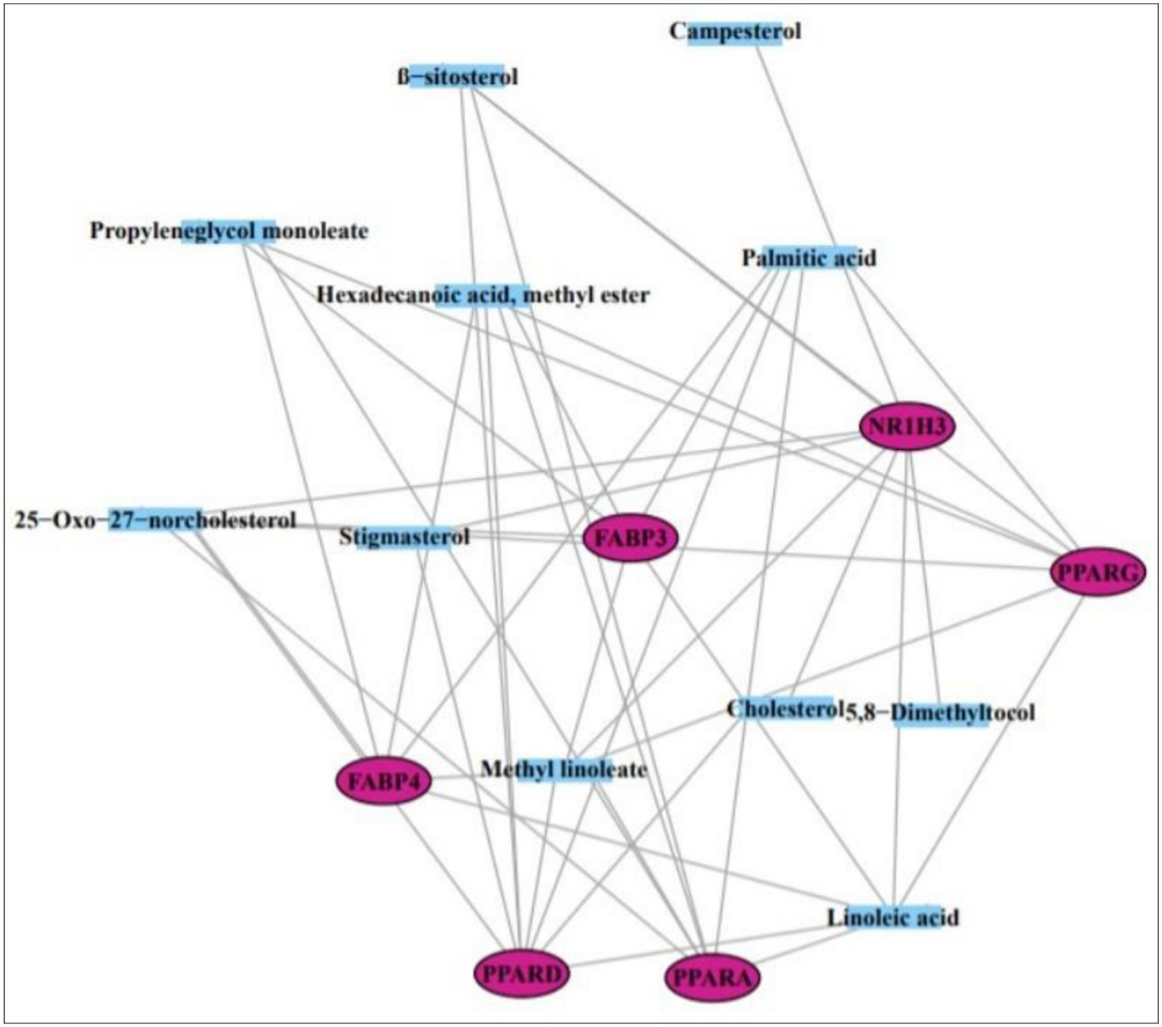
Interaction of 11 compounds and 6 genes on PPAR signaling pathway.

**Fig 8:**
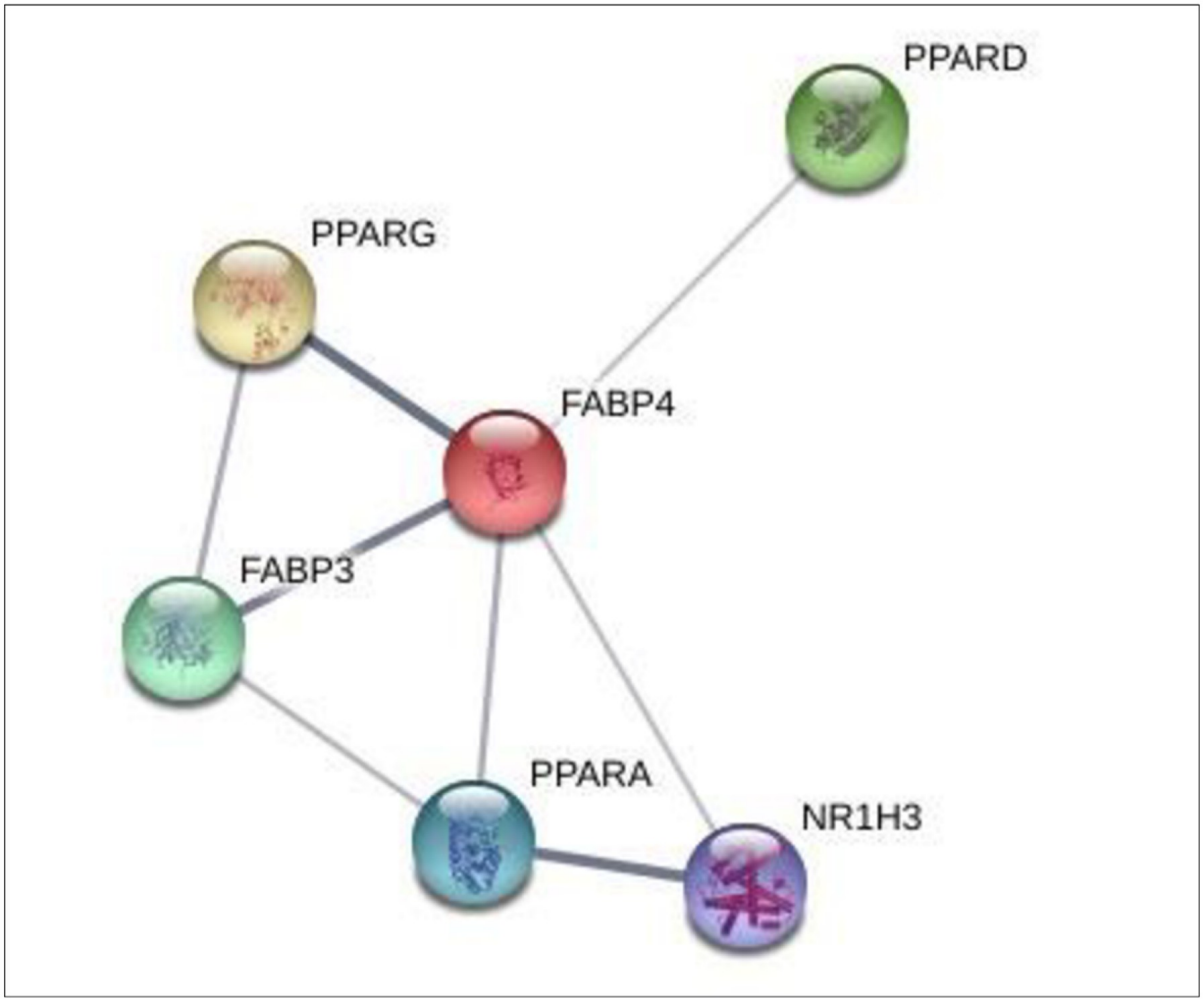
Interaction among 6 genes on PPAR signaling pathway.

From the SEA and STP databases, it was revealed that FABP3 gene is associated with six compounds (Methyl lineoleate, Linoleic acid, Hexadecanoic acid-methyl ester, Palmitic acid, Propyleneglycol monoleate, and 25-Oxo-27-norcholesterol), FABP4 gene is related to six compounds (Methyl linoleate, Linoleic acid, Hexadecanoic acid-methyl ester, Palmitic acid, Propyleneglycol monoleate, and 25-Oxo-27-norcholesterol), NR1H3 gene is involved with 8 compounds (β-sitosterol, Campesterol, 25-Oxo-27-norcholesterol, Cholesterol, Methyl lineoleate, 5,8-Dimethyltocol, Linoleic acid, and Stigmasterol), PPARA gene is related to 7 compounds (Palmitic acid, Hexadecanoic acid-methyl ester, Linoleic acid, Methyl linoleate, Propyleneglycol monoleate, β-sitosterol, and 25-Oxo-27-norcholesterol), PPARD gene is associated with 8 compounds (Hexadecanoic acid-methyl ester, Linoleic acid, Methyl linoleate, Stigmasterol, 25-Oxo-27-norcholesterol, Palmitic acid, Cholesterol, and β-sitosterol), PPARG gene is linked to 7 compounds (Methyl linoleate, Linoleic acid, Palmitic acid, Propyleneglycol monoleate, β-sitosterol, Hexadecanoic acid-methyl ester, and 25-Oxo-27-norcholesterol) (Fig 7). Also, each six proteins have strong molecular interactions on PPAR signaling pathway (Fig 8). The nominated compounds to dock with six proteins were selected via both SEA and STP.

**Fig 9:**
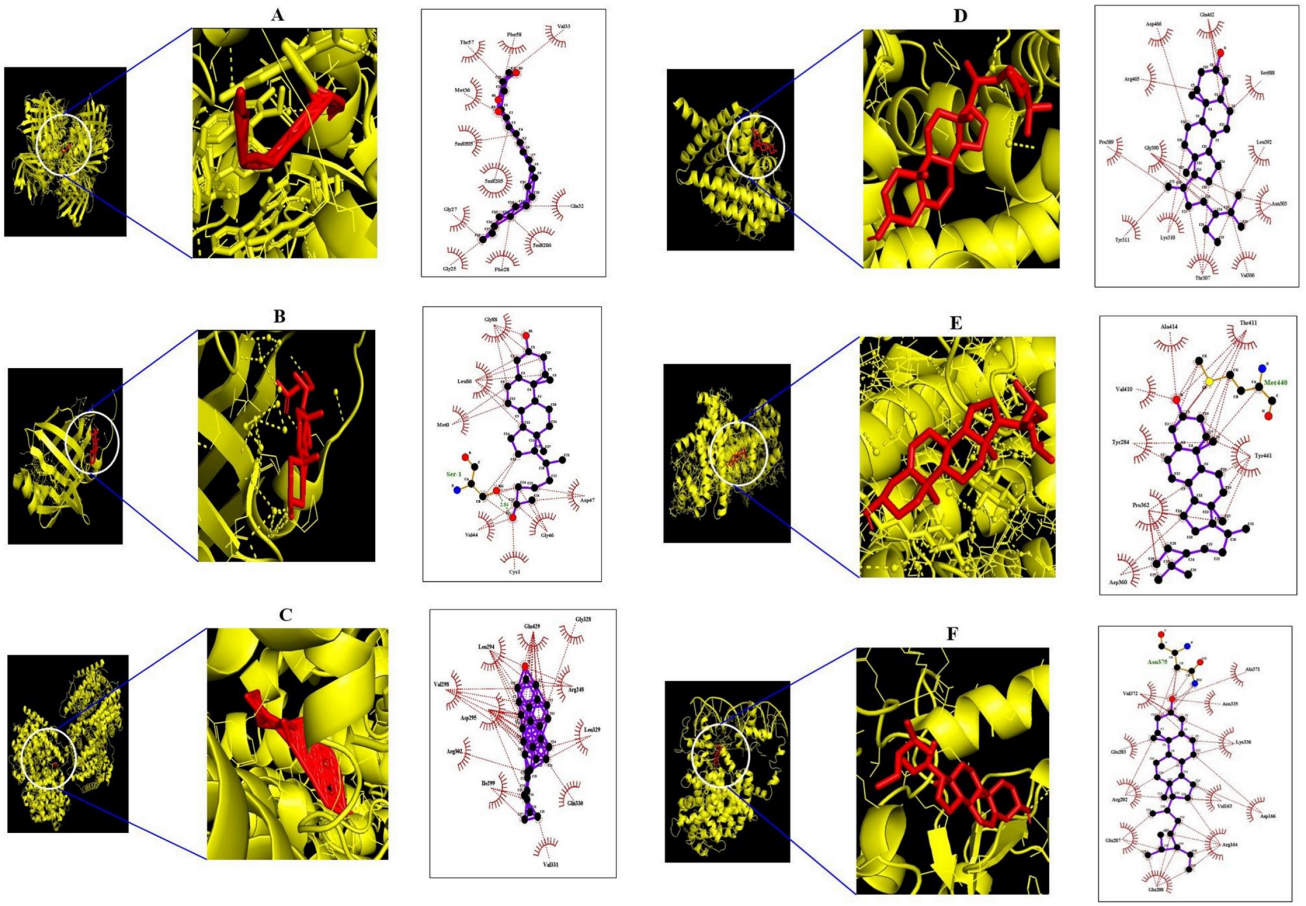
Molecular docking interaction between best docked compounds from SB and target proteins. (A) Propyleneglycol monoleate on 5HZ9 (B) 25-Oxo-27-norcholesterol on 3P6D (C) Campesterol on NR1H3 (D) β-sitosterol on 3SP6 (E) β-sitosterol on 5U3Q (F) β-sitosterol on 3E00.

**Fig 10:**
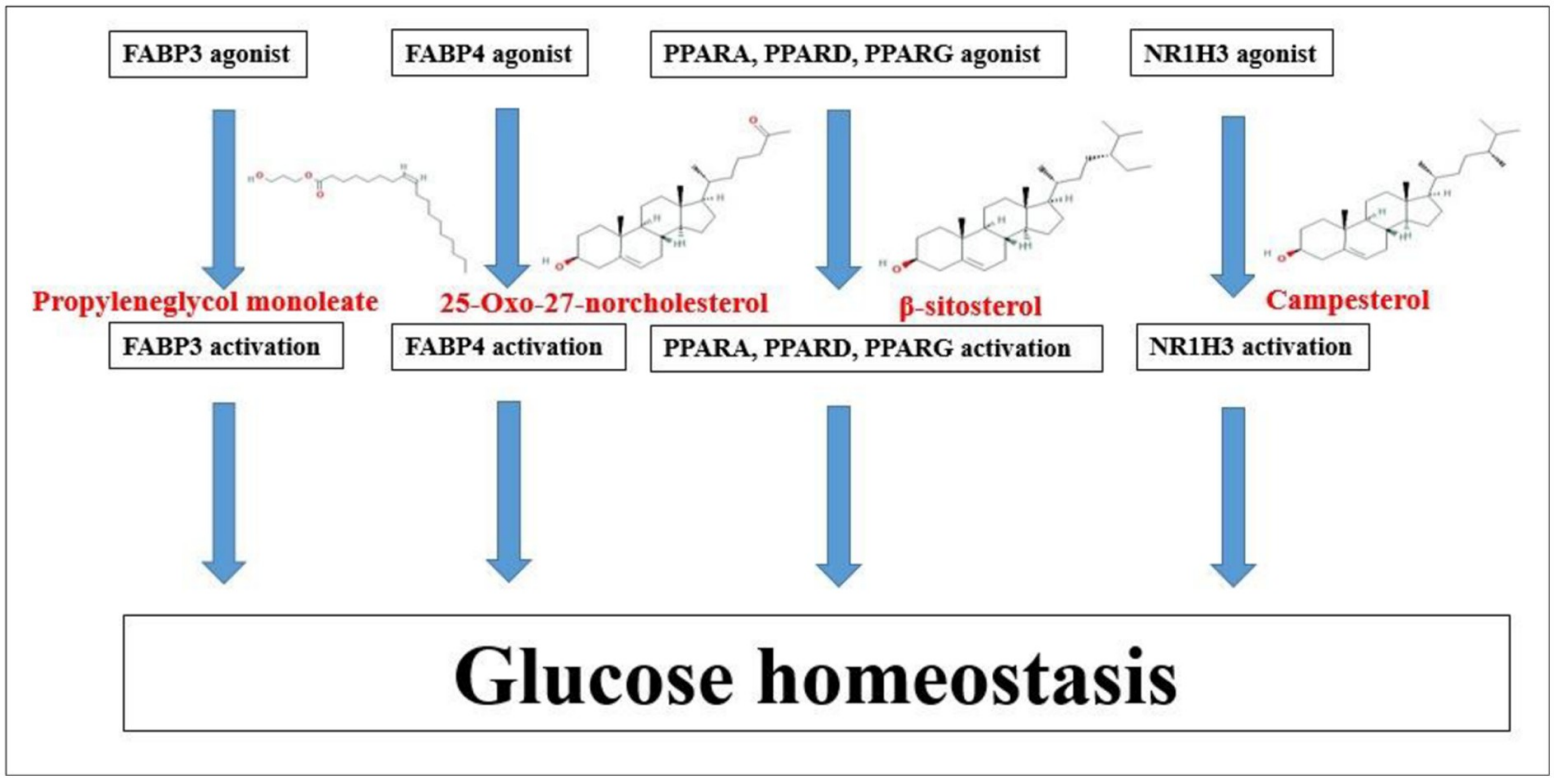
Regulation of glucose homeostasis by potential bioactive compounds of SB.

The molecular docking analysis was performed to evaluate the binding energy of these six genes against their related each gene, respectively, and the docking figures are depicted in Fig 9. Molecular docking score of A1-A6 on FABP3 protein (PDB ID: 5HZ9) is analyzed in the “*Homo Sapiens*” mode. Docking simulation of the affinity between A1-A6 and FABP3 protein in the “*Homo sapiens*” setting was analyzed. Based on the docking score, the order of the priority of binding energy is given: A5>A6>A2>A3>A1>A4. The six-binding energy of A1-FABP3, A2-FABP3, A3-FABP3, A4-FABP3, A5-FABP3, and A6-FABP3 demonstrated -6.6, -7.4, -6.7, -6.5, -8.4, and -7.8 kcal/mol, respectively. Interaction analysis of the best-docked compound namely “propyleneglycol monoleate” showed several hydrophobic bonds on FABP3 protein (PDB ID: 5HZ9). The hydrophobic amino acid residues are Thr-57, Phe-58, Val-33, Met-36, Gly-27, GLy-25, Phe-28, and Gln-32. The detailed information is enlisted in Table 4. The Propyleneglycol monoleate (A5) had the strongest affinity on FABP3. Docking simulation of the affinity between B1-B6 and FABP4 protein (PDB ID: 3P6D) in the “*Homo sapiens*” setting revealed promising binding affinity, and the order of the priority of binding energy is as follows: B6>B5>B2>B1>B3>B4. The six-binding energy of B1-FABP4, B2-FABP4, B3-FABP4, B4-FABP4, B5-FABP4, and B6-FABP4 revealed -4.6, -4.9, -4.5, -4.4, -5.6, and -7.2 kcal/mol, respectively. The 25-Oxo-27-norcholesterol (B6) had the strongest affinity on FABP4. Interaction analysis of best-docked compound namely “25-Oxo-27-norcholesterol” resulted one hydrogen bond (Ser-1) and seven hydrophobic bonds (Gly-88, Leu-86, Met-0, Val-44, Cys-1, Gly-46, and ASP-47). The detailed information is enlisted in Table 5. Docking simulation of the affinity between C1-C8 and NR1H3 protein in the “*Homo sapiens*” setting displayed promising binding affinity, and the order of the priority of binding energy is as follows: C2>C8>C3>C4>C1>C7>C6>C4. The eight-binding energy of C1-NR1H3, C2-NR1H3, C3-NR1H3, C4-NR1H3, C5-NR1H3, C6-NR1H3, C7-NR1H3, and C8-NR1H3 exhibited -7.3, -10.6, -7.9, -7.6, -4.9, -5.7, -5.9, and -8.3 kcal/mol, respectively. The campesterol (C2) has the strongest affinity on NR1H3. Interaction analysis of best- docked compound namely “campesterol” showed several hydrophobic bonds on NR1H3 protein (PDB ID: 2ACL). The hydrophobic amino acid residues are Gly-328, Arg-248, Leu-329, Gln-330, Val-331, Ile-299, Arg-302, Val-298, Asp-295, Leu-294, and Gln-429. The detailed information is enlisted in Table 6. Docking simulation of the affinity between D1-D8 and PPARA protein in the “*Homo sapiens*” setting revealed promising binding affinity, and the order of the priority of binding energy is as follows: D6>D7>D3>D5>D2>D1>D4. The eight-binding energy of D1-PPARA, D2-PPARA, D3-PPARA, D4-PPARA, D5-PPARA, D6-PPARA, and D7-PPARA exhibited -4.9, -5.2, -6.0, -4.8, -5.8, -6.6, and -6.1 kcal/mol, respectively. The β-sitosterol (D6) has the strongest affinity on NR1H3. Interaction analysis of best- docked compound namely “β-sitosterol” displayed several hydrophobic bonds on PPARA protein (PDB ID: 3SP6). The hydrophobic amino acid residues are Glu-462, Ser-588, Leu-392, Asn-303, Val-306, Thr-307, Lys-310, Tyr-311, Gly-390, Pro-389, Arg-465, and Asp-466. The detailed information is enlisted in Table 7. Docking simulation of the affinity between E1-E8 and PPARD protein in the “*Homo sapiens*” setting displayed promising binding affinity, and the order of the priority of binding energy is as follows: The eight-binding energy of E1-PPARD, E2-PPARD, E3-PPARD, E4-PPARD, E5-PPARD, E6-PPARD, E7-PPARD, and E8-PPARD exhibited -3.8, -5.2, -4.2, -7.3, -7.3, -4.6, -7.3, and -7.4 kcal/mol, respectively. The β-sitosterol (E8) has the strongest affinity on PPARD. Interaction analysis of best-docked compound namely “β-sitosterol” revealed one hydrogen bond (Met-440) and seven hydrophobic bonds (Ala-414, Thr-411, Tyr-441, Asp-360, Pro-362, Tyr-284, and Val-410). The detailed information is enlisted in Table 8. Docking simulation of the affinity between E1-E8 and PPARD protein in the “*Homo sapiens*” setting displayed promising binding affinity, and the order of the priority of binding energy is as follows: The seven-binding energy of F1-PPARG, F2-PPARG, F3-PPARG, F4-PPARG, F5-PPARG, F6-PPARG, and F7-PPARG exhibited -5.2, -5.4, -5.2, -5.9, -7.9, -4.0, and -7.8 kcal/mol, respectively. Interaction analysis of best- docked compound namely “β-sitosterol” exposed several hydrophobic bonds on PPARG protein (PDB ID: 3E00). The hydrophobic amino acid residues are Tyr-169, Tyr-189, Leu-167, Thr-168, Lys-336, Arg-350, Glu-351, Lys-354, Gln-193, and Tyr-192. The β-sitosterol (F5) has the strongest affinity on PPARG. The detailed information is enlisted in Table 9. This result suggested that each compound of the highest affinity score on each gene might be significant ligand to control glucose homeostasis (Fig 10).

**Table 4:** Binding energy and interactions of potential active compounds on FABP3 (PDB ID: 5HZ9).

					Hydrogen Bond Interactions	Hydrophobic Interactions
Protein	Ligand	PubChem ID	Symbol	Binding energy(kcal/mol)	Amino Acid Residue	Amino Acid Residue
5HZ9	Methyl lineoleate	5284421	A1	-6.6	n/a	Phe-58,Lys-22,
						Gly-25,Phe-28,
						Gln-32, Ala-29
	Linoleic acid	5280450	A2	-7.4	Lys-22	Ala-29,Gln-32,
						Phe-28, Gly-25,
						Gly-27
	Hexadecanoic acid, methyl ester	8181	A3	-6.7	n/a	Phe-28, Gln-32,
						Phe-58, Ala-29,
						Lys-22
	Palmitic acid	985	A4	-6.5	n/a	Val-33, Gln-32,
						Ala-29, Phe-58,
						Lys-22, Thr-57
	Propyleneglycol monoleate	5365625	A5	-8.4	n/a	Thr-57, Phe-58,
						Val-33, Met-36,
						Gly-27, Gly-25,
						Phe-28, Gln-32
	25-Oxo-27-norchoresterol	165617	A6	-7.8	Ser-35, Met-36	Asp-18, Phe-28,
					Met-36	Ala-29, Val-33,
						Asp-99, Gln-32
						Lys-10

**Table 5:** Binding energy and interactions of potential active compounds on FABP4 (PDB ID: 3P6D).

					Hydrogen Bond Interactions	Hydrophobic Interactions
Protein	Ligand	PubChem ID	Symbol	Binding energy (kcal/mol)	Amino Acid Residue	Amino Acid Residue
3P6D	Methyl linoleate	5284421	B1	-4.6	n/a	Ser-1,Cys-1
						Leu-86, Asp-47
						Leu-66, Ile-49
	Linoleic acid	5280450	B2	-4.9	Leu-86	Thr-85, Leu-66
						Asp-47, Cys-1
	Hexadecanoic acid, methyl ester	8181	B3	-4.4	n/a	Gly-88, Leu-86
						Ser-1, Gly-46
						Asp-47, Leu-66
						Met-0
	Palmitic acid	985	B4	-4.4	Glu-72,Val-80	Lys-79, Asp-71
						Val-73,Glu-61
						Thr-60
	Propyleneglycol monoleate	5365625	B5	-5.6	Gly-88, Leu-86	Asp-87, Met-0
						Ser-1, Cys-1
						Asp-47, Leu-66
						Ile-65
	25-Oxo-27-norchoresterol	165617	B6	-7.2	Ser-1	Gly-88, Leu-86
						Met-0, Val-44
						Cys-1, Gly-46
						Asp-47

**Table 6:** Binding energy and interactions of potential active compounds on NR1H3 (PDB ID: 2ACL).

					Hydrogen Bond Interactions	Hydrophobic Interactions
Protein	Ligand	PubChem ID	Symbol	Binding energy (kcal/mol)	Amino Acid Residue	Amino Acid Residue
2ACL	β-sitosterol	222284	C1	-7.3	n/a	His-395,Pro-396
						His-397, Gln-243
						Pro-242, Asp-398
						Ser-244, Glu-346
						Asp-241, Gln-348
						Asn-394
	Campesterol	173183	C2	-10.6	n/a	Gly-328, Arg-248
						Leu-329, Gln-330
						Val-331, Ile-299
						Arg-302, Val-298
						Asp-295, Leu-294
						Gln-429
	25-Oxo-27-norchoresterol	165617	C3	-7.9	Glu-339, Arg-404	Asp-379, Glu-390
						Ala-343, Asp-241
						Pro-242, Leu-347
						Pro-240, Glu-346
						Pro-386
	Cholesterol	5997	C4	-7.6	Asn-385	Trp-236, Ala-391
						Pro-237, Ile-238
						Glu-394, Pro-242
						Asp-241, Pro-240
						Glu-322, Lys-395
						Lys-326, Glu-388
	Methyl linoleate	5284421	C5	-4.9	Arg-342	Ile-238, Pro-378
						Ala-387, Pro-386
						Glu-390, Ser-411
						Phe-340, Asp-379
						Glu-339
	5,8-Dimethyltocol	222284	C6	-5.7	n/a	Leu-347, Pro-242
						Ser-244, Asp-241
						Glu-291, Gln-348
						Gln-243, Glu-346
						His-397, Asp-398
						Met-401
	Linoleic acid	5280450	C7	-5.9	Gly-328, Gln-422	Leu-329, Gln-330
						Gln-429, Asp-295
						Val-331, Lys-381
						Ile-299, Ala-303
						Arg-302, Glu-332
						Gly-382, Ala-425
						Val-298

**Table 7:** Binding energy and interactions of potential active compounds on PPARA (PDB ID: 3SP6).

					Hydrogen Bond Interactions	Hydrophobic Interactions
Protein	Ligand	PubChem ID	Symbol	Binding energy (kcal/mol)	Amino Acid Residue	Amino Acid Residue
3SP6	Palmitic acid	985	D1	-4.9	n/a	Glu-251, Val-332
						Ile-241, Ala-333
						Thr-279, Val-255
						Tyr-334, Leu-258
						Cys-275
	Hexadecanoic acid, methyl ester	8181	D2	-5.2	n/a	Ile-317, Ser-323
						Phe-218, Met-220
						Asn-221, Val-324
						Asn-219, Tyr-334
						Ala-333, Thr-279
						Leu-331, Leu-321
						Met-320, Thr-283
	Linoleic acid	5280450	D3	-6	Ser-323	Asn-221, Met-320
					Tyr-214	Val-324, Met-220
						Asn-219, Tyr-334
						Thr-279, Leu-331
						Leu-321, Thr-283
						Ile-317
	Methyl linoleate	5284421	D4	-4.8	n/a	Glu-286, Asn-219
						Gly-335, Tyr-334
						Thr-279, Val-324
						Leu-331, Leu-321
						Ile-317, Met-320
						Met-220, Phe-218
						Thr-283
	Propyleneglycol monoleate	5365625	D5	-5.8	n/a	Glu-251, Ala-250
						Val-255, Ala-333
						Met-220, Val-324
						Met-320, Tyr-334
						Thr-279, Cys-275
						Leu-254
	β-sitosterol	222284	D6	-6.6	n/a	Glu-462, Ser-688
						Leu-392, Asn-303
						Val-306, Thr-307
						Lys-310, Tyr-311
						Gly-390, Pro-389
						Arg-465, Asp-466
	25-Oxo-27-norchoresterol	165617	D7	-6.1	Lys-345	Asp-360, Glu-356
						Pro-357, Glu-439
						His-440, Leu-443
						Asp-353

**Table 8:** Binding energy and interactions of potential active compounds on PPARD (PDB ID: 5U3Q).

					Hydrogen Bond Interactions	Hydrophobic Interactions
Protein	Ligand	PubChem ID	Symbol	Binding energy (kcal/mol)	Amino Acid Residue	Amino Acid Residue
5U3Q	Hexadecanoic acid, methyl ester	8181	E1	-3.8	n/a	Pro-362, Tyr-284
						Arg-407, Glu-288
						Arg-361,Met-440
						Val-410, Thr-411
	Linoleic acid	5280450	E2	-5.2	n/a	Val-410, Arg-407
						Met-440, Asp-439
						Thr-411, Tyr-441
						Tyr-284, Asp-360
						Pro-362, Arg-361
						Glu-288
	Methyl linoleate	5284421	E3	-4.2	n/a	Arg-407, Glu288
						Tyr-284, Pro-362
						Met-440, Thr-411
						Val-410
	Stigmasterol	5280794	E4	-7.3	Met-440	Ala-414, Thr-411
						Tyr-441, Asp-360
						Pro-362, Tyr-284
						Val-410
	25-Oxo-27-norchoresterol	165617	E5	-7.3	Met-440	Ala-414, Thr-411
						Tyr-441, Asp-360
						Pro-362, Tyr-284
						Val-410
	Palmitic acid	985	E6	-4.6	n/a	Tyr-441, Pro-362
						Arg-361, Val-410
						Tyr-284, Glu-288
						Met-440, Thr-411
						Ala-414, Arg-407
	Cholesterol	5997	E7	-7.3	Met-440	Val-410, Ala-414
						Tyr-441, Tyr-284
						Asp-360, Arg-361
						Pro-362, Thr-411
						Val-410
	β-sitosterol	222284	E8	-7.4	Met-440	Ala-414, Thr-411
						Tyr-441, Asp-360
						Pro-362, Tyr-284
						Val-410

**Table 9:** Binding energy and interactions of potential active compounds on PPARG (PDB ID: 3E00).

					Hydrogen Bond Interactions	Hydrophobic Interactions
Protein	Ligand	PubChem ID	Symbol	Binding energy (kcal/mol)	Amino Acid Residue	Amino Acid Residue
3E00	Methyl linoleate	5284421	F1	-5.2	Tyr-169, Gln-193	Leu-167, Asp-337
						Lys-336, Val-372
						Lys-373, Glu-369
						Tyr-189, Thr-168
						Tyr-192, Arg-350
						Glu-351
	Linoleic acid	5280450	F2	-5.4	Thr-162, Leu-167	Arg-202, Asp-166
						Lys-336, Glu-369
						Glu-369, Val-372
						Arg-350, Glu-351
						Gln-193, Lys-354
						Tyr-192
	Palmitic acid	985	F3	-5.2	Ser-342, Glu-343	Leu-333, Arg-288
						Glu-291, Glu-295
						Met-329, Ala-292
						Pro-227, Phe-226
						Ile-341, Leu-228
	Propyleneglycol monoleate	5365625	F4	-5.9	Ser-332, Tyr-222	Lys-230, Phe-295
						Glu-295, Ile-296
						Ala-292, Arg-288
						Leu-333, Leu-228
						Met-329, Thr-229
	β-sitosterol	222284	F5	-7.9	n/a	Tyr-169, Tyr-189
						Leu-167, Thr-168
						Lys-336, Arg-350
						Glu-351, Lys-354
						Gln-193, Tyr-192
	Hexadecanoic acid, methyl ester	8181	F6	-4.0	Glu-343, Ser-342	Leu-340, Leu-228
						Ile-341, Met-329
						Phe-226, Ala-292
						Glu-295, Pro-227
						Arg-288, Leu-333
	25-Oxo-27-norchoresterol	165617	F7	-7.8	Asn-375	Asn-335, Lys-336
						Asp-166, Arg-164
						Glu-208, Glu-207
						Val-63, Arg-202
						Glu-203, Val-372

## 4. Discussion

Compounds-genes networking system unveiled that therapeutic effect of SB against T2DM was related to 16 compounds out of 20 compounds detected by GC-MS, including five steroid derivatives, four fatty acyls, two fatty acid esters, one organooxygen, one prenol lipid, one pyrimidine nucleoside, one azole, and one coumarin. The proportion of steroid derivatives to 16 compounds was close to 30%, implying that steroid derivatives was the most essential than any other sorts of compounds for the amelioration effect of SB on T2DM.

It was reported that some steroid derivatives have strong effect on hypoglycemic activity based on lipophilic properties [32]. Noticeably, a report showed that β-sitosterol (steroid derivatives) controls the glycemic level through regulation of IR (insulin receptor) and GLUT4 (glucose transporter 4) proteins in adipocytes of high fat and sucrose treated type 2 diabetic rats, interestingly, the *in vivo* result was in line with *in silico* analysis [33]. In addition, researchers found that campesterol (steroid derivatives) decreases the LDL (Low Density Lipoprotein) level, associated with the occurrence and development of T2DM [34, 35]. A patent revealed that propyleneglycol monoleate is an agent for treatment or amelioration of diabetes, obesity or arteriosclerosis [36]. Another report suggested that 25-Oxo-27-norcholesterol interrupts cholesterol oxidation, which is related to insulin resistance [37, 38]. These results coincide with our findings of SB on T2DM, suggesting that the quantity of the four compounds in SB is greatly enough to exhibit anti-diabetic efficacy.

Compounds-genes networking also specified that the pharmacological effect of SB on T2DM was directly associated with 81 genes. The results of KEGG pathway enrichment analysis of 81 genes showed that 12 signaling pathways were directly linked to the occurrence and progression of T2DM, demonstrating that these signaling pathways might be the key pathways of SB against T2DM. The 12 signaling pathways with T2DM were succinctly discussed as follows.

PPAR signaling pathway: PPAR ligands are the potential therapeutic candidates against T2DM, also, alleviate metabolic syndrome including obesity and insulin resistance [39]. Furthermore, dual agonists approach with both PPARA agonists (such as fibrates) and PPARG agonists (such as thiazolidinediones) can have better metabolic efficacy and less side effects than its single administration [40]. cAMP signaling pathway: cAMP signaling pathway modulates glucose homeostasis with insulin and glucagon secretion, glucose uptake, gluconeogenesis, glycogen synthesis and breakdown of glucose [41]. HIF-1 signaling pathway: Inhibition of HIF-1 signaling caused by diabetes is associated with hypoxia and high degradation of HIF-1α protein [42]. Sphingolipid signaling pathway: Sphingolipid is a significant class of signaling lipids, have been recognized as vital players in the progression and pathogenesis of insulin resistance and T2DM [43]. AMPK signaling pathway: The activation of AMPK enhances homeostasis of glycemic level, lipid concentration, and blood pressure in insulin-resistant rodents, which is considered as an important therapeutic target against T2DM [44]. VEGF signaling pathway: VEGF is related deeply to the development of T2DM. High content of VEGF is generally detected in plasma of T2DM patients [45]. T cell receptor activation: Overactivated T cells with T2DM patients might be an indication of losing the natural regulatory mechanism, thus, intervention of T cells by T2DM might down T cell receptor sensitization [46]. Fc epsilon RI signaling pathway: Fc epsilon RI- mediated signaling in mouse bone marrow is potentiated by insulin [47]. Estrogen signaling pathway: In a research of postmenopausal mice and human cells, the report demonstrated that estrogen is related to lower risk of T2DM by targeting particular cells in the pancreas and gut to improve tolerance to glucose [48]. Prolactin signaling pathway: A normal range of prolactin concentration is linked to a lower T2DM risk, which may play an inhibitory effect on the development of T2DM [49]. Adipocytokine signaling pathway: Adipocutokines leptin and adiponectin might be significant biomarkers for first prediction on T2DM, which is further associated with diabetic microvascular complications [50]. AGE-RAGE signaling pathway in diabetic complications: The activation of receptor for AGE (RAGE) is a noticeable pathological consequence on T2DM, and thus the design of antagonist for the AGE(RAGE) receptor might be a therapeutic strategy against T2DM [51].

Collectively, this study suggests that 12 signaling pathways with 81 genes are linked to the development of T2DM. In addition, rich factor defines that the proportion of the DEGs number and the number of genes have been annotated in pathway [52]. In other words, the higher of the rich factor is, the higher the degree of enrichment. The rich factor of PPAR signaling pathway was the highest degree among 12 signaling pathways. Reports indicated that PPAR agonists are insulin sensitizers and improve insulin resistance with T2DM patients [53]. To sum things up, a hub mechanism of SB against T2DM might be to maintain glucose homeostasis by activating PPAR signaling pathway.

## 5. Conclusion

*Sorghum bicolor* (SB) is rich in beneficial phytoconstituents and seen as a possible source of natural antidiabetic agents. However, in this report, its comprehensive bioactive compounds and T2DM pathways were firstly investigated using the network pharmacology. The findings of this study indicate that the antidiabetic ability of the SB could be attributed to four main compounds (β-sitosterol, campesterol, propyleneglycol monoleate, and 25-Oxo-27-norcholesterol) that were strongly related to PPAR signaling pathway. Therefore, our study suggests that the four key compounds of SB might ameliorate T2DM by activating the PPAR signaling pathway.

## Supporting information

S1 Table (PDF)

S2 Table (PDF)

S3 Table (PDF)

S4 Table (PDF)

S1 File (DOCX)

## Abbreviations

AMPK: AMP-activated protein kinase
cAMP: cyclic AdenosineMonoPhosphate
GC-MS: Gas Chromatography Mass Spectrometry
HIF-1: Hypoxia Inducible Factor-1
IR: Insulin Receptor
KEGG: Kyoto Encyclopedia of Genes and Genomes
LDL: Low Density Lipoprotein
MS: Mass Spectrometry
PPARA: Peroxisome Proliferator-Activated Receptor Alpha
PPARD: Peroxisome Proliferator-Activated Receptor Delta
PPARG: Peroxisome Proliferator-Activated Receptor Gamma
SB: *Sorghum bicolor*
SEA: Similarity Ensemble Approach
STP: SwissTargetPrediction
VEGF: Vascular Endothelial Growth Factor
