# Supplementary material for: Network pharmacology of bioactives from Sorghum bicolor with targets related to diabetes mellitus
Source: PLoS One. 2020 Dec 31;15(12):e0240873. doi: 10.1371/journal.pone.0240873 (PMC7774932; doi:10.1371/journal.pone.0240873)
Supplement: S1 Table — (PDF) [file pone.0240873.s001.pdf]

A list of 308 genes in SEA	A list of 324 genes in STP
AKR1B1	GBA
NCOA3	AKR1B1
P2RY6	ADA
P2RY2	CDA
SLC29A1	ADK
P2RY14	DPP4
P2RY4	ADORA1
SLC28A2	ADORA2A
CDA	ADORA3
ADK	HSPA8
ADORA3	GAPDH
HSPA5	AHCY
AMD1	HSPA5
HSPA8	TK1
ST6GAL1	MAPK1
ADORA1	MCL1
TOP1	PYGM
DNPH1	PDCD4
ADORA2A	CA2
P2RX1	CA1
RNASE1	TYMS
RNASE2	OGA
KMT5C	CA12
SETDB1	CA9
RNASEL	GAA
DNMT3B	GSK3B
GAPDH	FUCA1
SRM	PNP

P2RY11	PIM1
POLB	EHMT1
ADA	EHMT2
AHCY	CA14
GPR17	GRK1
SMS	CCND1
PARG	CDK4
TK1	DAO
GBA	F2
HEXA	IDO1
HEXB	CTRB1
CD69	NISCH
POLG	SLC6A3
QARS	PIK3CA
P2RX4	PIK3R1
ATIC	CTSK
DOT1L	CTSL
PLCG1	CTSB
ADORA2B	CYP1B1
RAC1	SIGMAR1
PDCD4	CHRM2
SLC5A2	CHRM1
KMT2A	CYP19A1
EHMT1	MAOB
CA9	BRD2
TPMT	TBXAS1
CA12	UTS2R
EZH1	GABRB3
SUV39H1	GABRG2

CA14
P2RY1
CDC42
SETD7
HPRT1
CA1
POLA1
FGF1
OGA
DNMT1
PAX8
TYMP
TK2
PNP
FGF2
ERAP1
OGT
GRK1
CA2
LGALS9
INMT
LGALS3
LGALS1
EHMT2
MAP3K7
HSPA1A
PIN4
PAM
LPAR4
GABRA5
DRD4
MAPK14
CHRM4
CHRM5
CHRM3
MGLL
CCR5
FABP4
FABP3
PTGS1
HSD11B1
PPARG
CNR1
FFAR1
FAAH
PTGS2
PTPN1
PPARA
FABP5
FABP1
CES2
CYP17A1
PPARD
CNR2
SCD
NOS2
TNKS
PTGES

LPAR6	NR1H3
WRN	HRH3
TNF	NPY5R
CES2	ALOX5
LPAR3	HRH4
PPP1CC	CYP11B1
PPM1B	CYP11B2
CES1	IMPDH2
PPID	HTR2A
TLR2	OPRD1
PRNP	ROCK2
LPAR1	GCK
P2RY10	GRIN1
MPEG1	HTR6
PLA2G2C	C5AR1
GPR174	QPCT
SLC25A20	GABRA3
GPR34	GABRA1
POLM	GCGR
PLA2G4B	GRIN2B
GNAI1	GABRA2
GNAI3	SLC6A9
DNM1	LIMK2
ADH7	HTR2C
ACER2	CALCRL
POLH	GPR119
COL4A3BP	SRC
FAAH	KIF11
LPAR2	EPHB4

PLA2G5	ALOX5AP
EPHX1	ASAH1
POLK	CRHR1
GNAO1	CDC7
FDPS	ABL1
KDM5A	YES1
GGPS1	LCK
PAOX	AURKB
PRKCA	PFKFB3
EPHX2	SLC9A1
SMPD2	JAK2
ADH1B	PARP2
ENPP2	RAMP1
ACP1	FLT1
SPHK1	APH1B
NAAA	PAM
POLL	SIRT2
S1PR2	PRKCA
KAT2B	PRKCQ
CDC25B	PRKCH
HAO1	PRKCG
PLA2G4C	UGT2B7
CNR1	HMGCR
NOD1	PRKCE
PLA2G2A	CPT1A
FABP3	VDR
GBA2	OPRK1
THRA	POLA1
ADH1C	AKT1

PTPN13	PRKCD
THRB	PREP
FUT7	AR
CDC25C	HSD17B2
ADH1A	SERPINA6
IARS	PRSS1
CNR2	TRPV1
VEGFA	SHBG
ASAH1	AKR1C3
HSD17B3	AKR1C2
DAGLA	AKR1C1
TRPV1	ABCB1
SLC22A8	NR3C1
HMGCR	DNM1
PGA5	ACACB
S1PR3	MET
PTPRC	LPAR6
SLC22A6	LPAR5
RARB	NLRP3
PLCG2	BACE2
PPARG	CTSD
SELP	S1PR3
TOP2A	S1PR1
LPAR5	PTPN2
APEX1	ENPP2
PPARA	G6PD
S1PR4	ESR2
CDC25A	NPC1L1
CA3	ESR1

CA7	PTPN6
GPR84	PTGER1
BBOX1	PTGER2
OXER1	CYP51A1
CYP4F2	CDC25A
GABRQ	RORA
KDM7A	CDC25B
PHF8	RORC
GSTK1	BCHE
BHMT	BACE1
SLCO2A1	PTGER4
SELL	PTGIR
SLC6A11	HSD11B2
LTB4R	PGR
GSTM1	FNTA FNTB
KDM2A	PTPN11
KDM4A	AKR1B10
KDM4C	POLB
PTGER2	PTPRF
GSR	PLA2G1B
TBXAS1	ACP1
ENPEP	SREBF2
PLA2G10	TERT
S1PR5	FDFT1
TBXA2R	SLC6A4
FOLH1	NR3C2
PTGER4	CYP2C19
PTGIR	CD81
PLA2G4A	TOP2A

FFAR4	PTGFR
SLC22A1	PTGER3
GSTA1	SLC22A6
GABBR2	TOP1
GABBR1	SLC6A2
PTGER3	PTGDR
FABP4	SAE1 UBA2
PTGFR	SRD5A2
LAP3	ACHE
GPR35	NR1H2
ATG4B	NR1I3
KDM4E	GLRA1
GABRR1	SQLE
PPARD	DHCR7
S1PR1	DRD2
KDM5C	SHH
CPT2	PHLPP1
PAFAH1B2	ILK
KAT5	PSEN1
SPTLC1	AURKA
SPTLC2	CCKAR
Ephx1	PDK1
MGLL	PDE10A
PRKCE	HTR1A
ABCC2	STS
SOAT2	CTSS
ALOX5	JAK3
FFAR1	JAK1
ALOX12	FASN

SPHK2	TACR2
STS	TACR1
CYP17A1	HTR1B
NPC1L1	HTR1D
GPBAR1	MCHR1
SRD5A2	MDM4
SLC10A1	MDM2
G6PD	CA7
SHBG	CA3
SERPINA6	CA6
SRD5A1	CA4
ABCC4	CA13
CD4	CA5B
CDC45	CA5A
GC	NFKB1
ST3GAL1	MAOA
VDR	NQO1
NR1H3	XDH
NR1H4	EGFR
AR	FABP2
ESR2	PHF8
RORA	NR1H4
CYP24A1	HSD17B3
SLC10A2	GABBR1
ABCB11	KDM2A
CYP19A1	KDM5C
SREBF2	GPBAR1
ESR1	GABRB2
GABRD	HAO1

EBP
EPHA7
CYP27B1
EPHA2
EPHA5
EPHA4
EPHA8
EPHB3
EPHB1
EPHA1
EPHA6
SLC22A3
SHH
EPHB2
RORC
UGCG
EPHB6
ABCB1
NR1H2
PHLPP1
TTPA
AKT1
IL2
Ar
CA5B
CA6
CA5A
CA4
KCNA7
GSTK1
LTA4H
NR0B2
CDC45
PTPRC
RXRA
CYP26A1
CYP26B1
RARG
RARB
RARA
FFAR4
CACNA2D1
RXRG
ALOX15
ALOX12
RXRB
SLC16A1
ABCC1
EPHX2
CHRNA7
PLG
TBXA2R
PLA2G4A
HTR2B
RORB
UBA2
SAE1
GRM5

KCNA1
CA13
MAOB
KCNA4
NQO1
KCNA2
KCNA3
PCSK7
PGR
AHR
KCNN1
NFKB1
CYP1B1
MAOA
HNF4A
KCNN2
DRD4
MIF
KCNA5

HNF4A
LTB4R
PDE4D
SLC22A12
PTGDR2
IL6
GLUL
ADRA2B
EDNRA
MAPK3
TRPM8
APH1A
NCSTN
PSENEN
PSEN2
CMA1
CTSG
GRM2
OXER1
PDE4A
RBP4
METAP1
MMP2
PTGES2
PRKAA2
PRKAB1
PRKAG1
PDE4B
ITGB2

ICAM1
ITGAL
MPEG1
SLC1A1
F2R
DCK
