# Supplementary material for: Network pharmacology of bioactives from Sorghum bicolor with targets related to diabetes mellitus
Source: PLoS One. 2020 Dec 31;15(12):e0240873. doi: 10.1371/journal.pone.0240873 (PMC7774932; doi:10.1371/journal.pone.0240873)
Supplement: S2 Table — (PDF) [file pone.0240873.s002.pdf]

A list of 4,736 genes related to Type2 diabetes

D7CNTP
ALPQTL2
ANON1
BCC1
BMND3
MLM
DNS
CMM
CCV
CTRCT8
DYX8
HLM2
ERPL1
IBD7
MS4
MYP14
PSORS7
PTPRZ2
MTS1
TFS1
SAI1
MRS
SAMD11
NIR
NOC2L
HES4
IMD38
IFI15

G1P2
ISG15
CMS8
AGRN
GITR
AITR
TNFRSF18
TNFRSF4
TXGP1L
OX40
ACT35
IMD16
CAB45
SDF4
B3GALT6
ALGAZ
EDSSPD2
SEMDJL1
C1QTNF12
C1QDC2
FAM132A
SCNN1D
RC68
INTS11
CPSF3L
CPTP
GLTPD1
T1R3
TAS1R3

DRS2
DVL1
ASP3
MXRA8
CCNL2
MRPL20
WARP
VWA1
KIAA1273
TOB3
ATAD3B
PHRINL
ATAD3A
HAYOS
SCA21
C1orf70
TMEM240
SSU72
MIB2
MMP22
MMP23B
CDK11
P58
CDC2L1
CDK11B
MMP23A
MMP21
MIFR
CDC2L2

CDK11A
NADK
GNB1
MRD42
GABRD
GEFSP5
EIG10
EJM7
PKC2
PRKCZ
C1orf86
FAAP20
SGS
SKI
KONDS
PEX10
NALD
PBD6A
PBD6B
PLCH2
PLCL4
PANK4
HES5
TR2
HVEM
TNFRSF14
SEP
NL1
NEP2

NEPII
MMEL1
ARPM2
ARPT2
ACTRT2
CMD1LL
LVNC8
MEL1
PRDM16
EGFL3
MEGF6
FAM79A
TPRG1L
TP73
VEL
SMIM1
JBTS25
KIAA0562
GLYBP
CEP104
DFNB96
SLSN4
NPHP4
KCNA2B
KCNAB2
CHD5
EAP
RPL22
C1orf188

RNF207
ICMT
GPR153
BACH
LACH
ACOT7
HES2
ESPN
USH1M
TNFRSF25
TNFRSF12
DR3
LARD
PLEKHG5
CMTRIC
DSMA4
KIAA0720
T1R1
TAS1R1
HKR3
ZBTB48
CANPMR
KIAA0833
CAMTA1
GLC3B
SCZD12
VAMP3
FASPS3
PER3

CD137
ILA
TNFRSF9
PARK7
DJ1
RALT
MIG6
IDDNPF
DNB5
SLC45A1
RERE
NEDBEH
ENO1
PPH
MPB1
CA6
SLC2A7
GLUT7
SLC2A5
GLUT5
H6PD
GDH
G6PDH
CORTRD1
SPSB
SSB1
SLC25A33
PIK3CD
APDS

IMD14
CLSTN1
KIAA0911
CTNNBIP1
ICAT
LZIC
NMNAT1
NMNAT
PNAT1
LCA9
UBE4B
UFD2A
KIAA0684
KIF1B
CMT2A
CMT2A1
NBLST1
PGD
APITD1
CORT
DFFA
DFF1
PEX14
PBD13A
CASZ1
SRG
TARDBP
TDP43
ALS10

MASP2
SRM
SPS1
EXOSC10
PMSCL2
MTOR
FRAP1
SKS
ANGPTL7
CDT6
UBIAD1
TERE1
SCCD
DISP3
PTCHD2
KIAA1337
FBXO2
FBX2
FBG1
NFB42
FBXO44
FBX44
FBX6A
FBG3
FBXO6
FBX6
FBG2
MAD2L2
MAD2B

FANCV
MTHFR
CLCN6
NPPA
PND
ANP
ATFB6
NPPB
BNP
PLOD1
LH1
LLH
EDSKCL1
MFN2
KIAA0214
CMT2A2A
HMSN6A
MIIP
IIP45
TNFRSF8
CD30
D1S166E
TNFRSF1B
TNFR2
TNFBR
VPS13D
SCAR4
GBD2
DHRS3

RETSDR1
LRRC38
PDPN
TI1A
T1A2
GP36
PRDM2
RIZ
KAZN
EFHD2
SWS1
CTRC
CLCR
CELA2A
ELA2A
AOMS4
CELA2B
ELA2B
CASP9
APAF3
AGMAT
AUH
RSC1A1
RS1
PLEKHM2
SKIP
KIAA0842
FBLP1
MIGFILIN

ATRST2
SPEN
MINT
SHARP
HIAA0929
ANIB3
EA8
ZBTB17
ZNF151
MIZ1
CMT2A2B
HSPB7
CVHSP
CLCNKA
CLCNKB
EPHA2
ECK
CTPP1
CTPA
FBXO42
FBX42
KIAA1332
NECAP2
NBPF1
KIAA1693
RNU1A
RNU1
TRNAE1
TRNE

TRE
OTS8
AGGRUS
CROCC
ROOTLETIN
KIAA0445
MFAP2
MAGP
MAGP1
ATP13A2
PARK9
KRPPD
SPG78
SDHB
SDH2
SDHIP
PGL4
PADI2
PADI1
PADI3
UHS1
PADI4
PADI5
PAD
PADI6
PREMBL2
ARHGEF10L
GRINCHGEF
KIAA1626

PAX7
RMS2
MYOSCO
TAS1R2
T1R2
ALDH4A1
ALDH4
P5CDH
UBR4
ZUBR1
RBAF600
KIAA1307
EMC1
KIAA0090
CAVIPMR
ARCC2
CTRCT6
AKR7A3
AFAR2
AKR7A2
AKR7
AFAR
PQLC2
CAPZB
CAPPB
MICOS10
MINOS1
MIO10
MIC10

D1S1733E
DAN
HTR6
RNF186
OTUD3
KIAA0459
PLA2G2E
PLA2G2A
PLA2B
PLA2L
MOM1
PLA2G5
FRFB
BFIS4
BFIC4
PLA2G2D
SPLASH
PLA2G2F
UBXN10
MUL1
MULAN
C1orf166
CDA
PINK1
PARK6
DDOST
OST
OST48
CDG1R

KIF17
KIAA1405
INS
PPARG
CP
RAC1
APPL1
EIF2S3
FN1
SIRT1
IRS1
LEPR
ATP6
POMC
PON1
PTGS2
SOD1
CAT
NR1I2
ADIPOQ
AOC3
IL2RA
PAX6
CYBB
NR1I3
MAP3K5
MT2A
NCF1
CPT1A

MAFA
ALPK1
KIF1A
THBS2
ADRB1
GAD1
GLP1R
ZFP57
KIF5B
SERPINE1
PPARA
NR0B2
CLOCK
PTEN
ARNTL
ABCG2
ICA1
PROM1
SLC22A12
LRP1
CDKN2A
SLC19A2
CFTR
GLIS3
F7
FXN
PTPN22
GCK
GCKR

HFE
HLA-DQB1
HLA-DRB1
HNF4A
APOE
IGF1R
IL6
AQP1
AQP4
INSR
PDX1
KCNJ11
LMNA
LPA
ASIP
PAX4
IFIH1
STAT3
ABCC8
HNF1A
HNF1B
TCF7L2
TP53
UMOD
WFS1
FTO
CASR
APOA5
FOXP3

HAMP
CDKN2B-AS1
ATM
HLA-B
COX2
CISD2
BRCA1
GATA6
LIPC
BMP2
CAV1
PNPLA2
SPINK1
CUBN
DNM1L
GATA3
GJA1
ARG1
TRNL1
MLXIPL
PRKAR1A
DNAJC3
WRN
CNBP
ELN
BSCL2
SLC25A4
AIRE
ITPR3

KRAS
LIPE
ZFHX3
IER3IP1
PLIN1
BLK
AGPAT2
CPS1
FOXC2
FOS
PTF1A
GNAS
HBB
NDUFS7
SMAD4
COX1
CYTB
ND1
ND5
PRKAG2
PDE4D
PIK3R1
SLC29A3
PRSS1
SLC22A2
BRCA2
TLR1
C2
SLC30A3

DCAF17
MKKS
HS6ST1
FADS2
ADA
ZGLP1
HPN-AS1
CERT1
LIPC-AS1
LOC102723407
ADAR
RNASEH2A
TXNIP
CETP
HPSE
PPARGC1A
TRIM31
CAPN10
POLG2
TREX1
NLRP3
NDUFA11
GJB4
RBM45
PLEKHH2
CPA1
CRP
PARP1
MAPK14

CST3
HJV
CCN2
CTLA4
CTNNB1
ADRB3
CYP2C19
CYP21A2
DAB2
DBP
ACE
DECR1
SLC30A8
DKC1
AGER
DPP4
ATN1
AGT
DSPP
AGTR1
ABCA1
EDN1
CELSR2
AHSG
UBR1
PDILT
EPO
AKT1
ALB

FABP4
ALDH2
FGF2
PALLD
FOXO1
AKR1B1
SIK3
ZFYVE26
LEMD3
PLA2G15
TMEFF2
KDSR
GABPA
PCSK9
WDR72
GAD2
NDUFAF3
SAMHD1
COPD
ZBTB20
TINF2
GATM
FGF21
GCG
GCGR
GH1
GIP
GJB3
GLO1

GLUL
ABO
OR10A4
CAVIN1
NSMCE2
GPT
GPX1
NDUFAF4
CD274
GSK3B
GSTM1
GRHL1
HGF
HIF1A
HLA-A
HLA-DQA1
HMGB1
HMOX1
HP
HPN
APOA1
IAPP
APOB
ICAM1
IDE
GOLGA6A
APOC3
IFNG
IGF1

IGFBP3
APRT
IL1A
IL1B
IL1RN
IL4
CXCL8
IL10
IL17A
IL18
ITPK1
KCNQ1
LCN2
LDLR
LEP
LGALS3
LHX1
FADS1
LINC02694
LPL
LRP6
ARSA
MBL2
MC4R
MFAP1
MMP2
MMP9
MPO
TRNC

COX3
ND2
ND3
ND6
TRNF
TRNK
TRNQ
TRNS1
TRNS2
TRNV
TRNW
NDP
NDUFA1
NDUFA6
NDUFB3
NDUFB9
NDUFB10
NDUFS1
NDUFS2
NDUFS3
NDUFV1
NDUFS4
NDUFS6
NDUFS8
NDUFV2
NEUROD1
NFATC1
NFE2L2
NGF

NHS
NOS3
NPY
ATP2B1
OPA1
TNFRSF11B
RRM2B
NOX4
NEUROG3
PARN
PDE11A
TPRKB
ADIPOR1
NDUFAF1
TIMMDC1
POLK
ENPP1
RTEL1
SERPINF1
PEX1
PEX6
PIK3CA
PIK3CB
PIK3CG
PLCG1
PLG
PMM2
POLD1
POLG

SEMA5B
CCHCR1
NDUFB11
BCAS3
CDKAL1
SARS2
AVP
WRAP53
RCBTB1
CHDH
NOP10
SLC30A10
MAML3
FOXRED1
ZCCHC8
CARMIL1
PRKAA1
PRKAA2
PRKAB1
NHP2
PRKACA
TMEM126B
ACSS2
ANGPTL8
MAPK1
MAPK8
PRSS2
TWNK
RETN

PTH
SHROOM3
SUGP1
BBS1
BBS2
MOK
BCHE
ACE2
RBP4
REN
RENBP
ROS1
BDNF
BGLAP
CCL2
CXCL12
ELMO2
CDH23
SLC39A8
SI
ST3GAL4
SLC2A1
SLC2A2
SLC2A4
SLC5A1
SLC5A2
SLC34A1
SOD2
SPP1

TERC
TGFB1
TLR4
TNF
TTPA
C9
UCP1
UCP2
UCP3
VEGFA
BEST1
TRPV1
XRCC4
CAD
NDUFAF5
CCDC28B
RNASEH2B
USB1
PALB2
ARMC5
EHMT1
CTC1
NUBPL
PNPLA3
THSD7B
HMGA2
OR2B2
OR12D3
GPR101

CASP3
PIP5K1B
ARL6
RNASEH2C
ACCS
LMNB2
PDE8B
IRS2
ALDH1A2
ZPR1
MGAM
SELENBP1
BAZ1B
AIP
USP8
UBXN11
NDUFAF2
UBE2Q2
KL
FOXP2
EIF2AK3
CD34
CD36
GDF15
RAPGEF5
ADAMTS13
CYP2E1
CYP3A4
EGFR

ALOX15
GHR
IFNA1
IFNA13
P2RX7
P2RY12
SST
TAC1
VWF
PDHX
FGF23
ATP6AP2
AHSA1
IGF2BP2
UTS2
ADM
CRK
AFP
DMPK
FABP2
SIRT3
DIANPH
RNF19A
POLDIP2
HPGDS
ANGPT1
GSR
HHEX
HSD11B1

HSPD1
APP
MALAT1
SOST
BCL2
SREBF1
TCF7
TRBV20OR9-2
CALCA
AIMP2
KLF11
INSM2
SOCS3
GRAP2
ADH1B
DDIT3
EGR1
F3
RFX6
GAST
MSTN
GFAP
GSTT1
IGF2
IGFBP2
KLK3
LAD1
MME
HSPA14

PLAT
PPARD
PYY
PTPN6
G6PC2
PTPRN
REG1A
S100A8
S100B
SELE
SGK1
TLR2
TRPM2
VCAM1
PLA2G7
ADIPOR2
APOL1
TNFSF10
CCK
APLN
CD59
C20orf181
AKR1A1
CEL
OGA
NQO1
DMD
FBL
F2

PTK2B
G6PC
GNB3
ANGPT2
ANPEP
HBA1
AOC2
FOXA2
HSPA5
IL2
CXCL10
KDR
KNG1
LTA
MAP6
MAPT
NFKB1
NOS2
ACR
IL22
SERPINA5
SIRT6
PDK4
PLXNA2
PRKCB
SCD
SOAT1
SPARC
CCR2

VEGFC
XBP1
CXCR4
RUNX2
SOCS1
HDAC3
NTN1
FGF19
NAMPT
PERCC1
SORBS1
MTCO2P12
PSIP1
SORCS1
CCR5
CNR1
PPARGC1B
CPOX
CTSD
CUX1
CYBA
ADRB2
DPT
AGRP
ERBB2
JAZF1
MMRN1
CLEC16A
ANGPTL2

SMUG1
AMBP
SLC17A5
HAVCR1
SIGLEC7
ANGPTL3
IFNL3
BTBD8
NR3C1
NRG1
HSPA4
HSPB1
HSP90AA1
APOA4
IGFBP1
INPPL1
ISG20
JAK2
GSTK1
MET
ATF4
NUCB2
OLR1
P4HB
PAEP
PCK1
PCK2
PECAM1
PRKCA

APOM
MAPK7
B2M
PTPRN2
PTX3
ACTB
S100A1
SORT1
S100A9
CCL5
SFRP5
BMP7
BTF3P11
TFPI
TFRC
THBS1
TSPO
TIMP1
DHX40
SPX
CNDP1
TNFSF11
PDE5A
BECN1
TRMT10A
ENTPD1
CD68
NAT2
LINC01672

MICA
ABCB6
ERVK-18
TRIM13
CEACAM5
CECR
RN7SL263P
CHGA
WDHD1
TREH
FBXO32
CLU
SLCO6A1
CREBBP
GPR119
CSF2
CSF3
CYP2C9
GRK2
CYP2J2
CYP11B2
DNMT1
JAG1
AGTR2
EDNRA
AHR
AKT2
ERN1
ACSL1

ATF6
SIRT2
DKK1
FOXM1
FLNA
NR5A2
GAP43
GAPDH
GFER
GGT1
GIPR
NOX1
GOT2
SETD2
GSTP1
ANXA1
HCLS1
HCRT
HK2
HLA-C
MNX1
HMGA1
AGFG1
HSPB2
IARS1
HCAR2
CCN1
IL2RB
KLK1

SUMO4
LGALS1
LOX
LRP2
SMAD2
SMAD3
MGP
MMP1
MMP3
MMP14
NOTCH1
NPPC
REG3A
PRKN
ANGPTL4
ADA2
ACP1
PON2
POR
SELENOS
MAPK3
TRIB3
BAX
S100A12
SELP
FN3K
HHIP
SLC6A2
SLC9A3

SNCA
STAT5B
STK11
BRS3
TCF3
TH
TKT
TM7SF2
TNFRSF1A
TRAF6
TRPC6
TTR
TXN
UCHL1
VPS51
NPHS2
NOX5
PPP1R3B
KMT2D
CALR
FZD4
CASP8
RPAIN
TRIM63
OGT
DEGS1
KHSRP
PEA15
RIPK1

HSPB3
ARTN
IL33
PPIG
CD38
CARTPT
ABCG1
NOS1AP
DCAF1
DGCR2
FOXO6
KIR2DS2
SH2B3
PPIF
PGR-AS1
CDK4
CDKN1C
TUBB4B
FST
ADCY5
CHI3L1
PRRT2
CHRM3
TNDM1
C1QTNF5
CMKLR1
CNTF
COL9A3
COMT

COPA
ADORA1
CPE
TRPM6
CSN2
NRG4
CTSB
ADRA2B
CTSS
CX3CR1
CXADR
CYP2B6
CYP2D6
CYP7A1
CBLL2
SLC35G1
DCN
DLD
DYRK1A
EDNRB
ELANE
ELK3
ENO2
EPHB1
EPHB2
ETV3
FLT1
CELA3B
CABIN1

DDAH2
DDAH1
CD2AP
AMPD1
DESI1
SGSM3
GNAO1
PSAT1
CFH
ACACA
HLA-DPB1
HLA-DQB2
HRC
HSPA1A
HSPA1B
IDDM7
IFNAR1
IL6R
IL15
INSRR
ITGAM
ITGB2
KCNMA1
KIR2DL2
LNPEP
ARR3
MEN1
MIP
MMP8

MT1A
MYD88
CCN3
ATP2A2
OGG1
PC
GAL
GP6
PCSK1
PDCD1
ISYNA1
IL23A
PDR
ARID4B
PGF
PGM1
SERPINA1
PIN1
PKD1
PLA2G1B
UBASH3A
PLTP
TLR9
PPIA
TET2
PPID
SLC52A1
PPP1R3C
SLC47A1

IMPACT
MEG3
CISD1
PRL
PROX1
SLC2A9
ACKR3
PSMD9
PTHLH
CIP2A
RAF1
IL21
RARRES2
TRPV4
RYR2
CFB
BGN
CCL4
CCL21
CXCL5
SELL
SELENOP
SLC2A3
SLC6A3
CXADRP1
SLC6A4
GGTLC5P
SLC22A1
SMS

SPG7
SYP
ZEB1
TEK
TFAM
TFE3
TIMP2
TIMP3
TIMP4
TNNI3
GGTLC3
GGT2
TYK2
GGTLC4P
VIM
ZFP36
MANF
ST8SIA4
HDAC11
ZC3H12A
SLC2A10
CANX
CAPN1
CASP1
PLA2G6
KAT2B
CCKAR
CDC123
MUC16

CD86
CD40
SOX13
CD40LG
CD44
BMS1
KEAP1
ELMO1
TMX2-CTNND1
CELA3A
TSHZ1
CDK5
RIDA
LOC102724197
CDKN2B
KLF2
SEMA3A
NOD1
RACK1
NSA2
CEBPB
HYOU1
PRDX4
CCT2
SCGN
KHDRBS1
CXCR6
CETN1
NFAT5

TCFL5
CTSC
LILRB1
SLC22A7
KCNQ1OT1
CHAT
BTN2A1
PDAP1
DBA2
UCN3
C1QTNF3
SLC26A9
H3P10
H3P40
ADCYAP1
MRGPRX3
MRGPRX4
ADD1
NOXO1
ADH1C
COL1A1
TMEM18
COL11A2
COMP
KLF6
GPR151
ADORA2A
COX8A
ADORA2B

CPB1
CRH
CRMP1
BPIFA2
CRYZ
CSE1L
PWAR1
C2CD4A
CSTA
LYPD4
ADRA1A
ADRA2A
CTNND1
PLB1
CTSL
CREBRF
CYP1A1
CYP8B1
CYP27B1
CD55
SLC16A11
TMPRSS6
OXER1
DDT
DEFB1
DHPS
DIO2
DPYD
DPYS

DUSP1
APLNR
ECE1
EIF5A
CELA1
CRTC2
ZBTB7C
EPOR
ERBB3
ERBB4
ERCC2
EREG
ALAD
ALCAM
EZH2
F2R
F2RL1
F8
F10
F11
FABP1
FAP
FASN
TPCN2
FBN1
FDPS
GPRC6A
FES
FGFR4

FKBP4
FKBP5
FOXO3
FLG
FLII
LPIN1
FMO3
PES1
LPAR3
DAPK2
BACE1
NUP62
ALOX12
ALOX5
ALOX5AP
PRPF6
IL27
FRMD3
TXN2
POC1A
PAMR1
MRGPRX1
SH2B1
AMD1
GATA4
AMD1P2
GCH1
AMH
CBLIF

FETUB
GJA3
FOXD3
GK
INTU
IL17B
GCLC
GLRX
GPER1
GPR35
GPR42
GRN
TBK1
GTF2H1
GUSB
GYPA
GYPB
GYPE
GZMB
ANXA2
HAS2
HDAC2
ANXA6
HLA-DQA2
PRMT1
HSF1
XIAP
HSPA9
APOA2

GPIHBP1
PWAR4
IGFALS
IGFBP7
FAS
IL3
CXCR1
IL13
IMPDH2
IRAK1
ITPR1
JUN
JUND
ENHO
KCNJ1
KISS1
KRT5
KRT16
TBPL2
C2CD4B
LAMC2
LAMP2
LBP
LCK
LETM1
LIF
LIG4
LRP5
LINC01194

ARNT
LYZ
MAFD2
MAS1
MB
MC3R
MCL1
MDM2
MECP2
MEF2A
MEFV
MIF
MIAT
VN1R17P
GPR166P
MSMB
MST1
MSX2
MVD
MYC
MYH9
ATF3
NEFL
NFKB2
NFKBIA
NGFR
NIDDM2
NNMT
NOTCH3

NPHS1
SLC11A2
NRF1
ATP2B2
OGN
P2RX3
P2RY1
IL20
PAWR
PBX1
ASCC1
PCNT
DCTN4
CYB5R4
PCSK2
TLR7
PCYT1A
DTL
UFM1
GHRL
ATP5PF
PGC
PHB
SLC25A3
PLAG1
PMP22
ERRFI1
TREM2
PON3

KRT20
ATR
DLL4
TRPM7
ANO1
SOX6
STAP2
KDM3A
PAG1
ZNF395
PSPN
PROC
PROS1
TRPV5
PSMA6
FAM20C
CAMK1D
PSPH
JPH3
PTGS1
RAD1
PVR
PVT1
UBL5
LGR6
BCL2A1
RNASE3
BACH2
IGAN1

MBOAT4
RPS6
RPS6KB1
SALL1
SRL
CCL11
CCL20
SRR
CIDEC
NOD2
ABCG8
COP1
SFTPD
SHC1
EBF2
BMP4
SLC5A3
SLC6A8
BMP6
SLC11A1
SLC18A2
BMPR2
SMPD1
SNAI1
SNAP25
SOX2
SP1
TRIM21
SSTR4

SSTR5
ST2
STAT1
STAT5A
STX4
STXBP1
STXBP3
SULT1A1
BSG
BTC
SYN2
TACR1
KLF9
TAPBP
TAT
TBP
TCF21
TFF3
TG
TGM2
TLR3
TSPAN7
TSPAN8
TMSB4X
TPD52
TPO
C3
NR2C2
TSC2

TTN
TWIST1
UBE2I
UTRN
VDAC1
VGF
WNT1
YY1
YWHAZ
PTTG1IP
ZMYM2
AIR
ALMS1
TNDM
SLC52A2
CORO7
VTCN1
LIN28A
CALCR
NR4A3
COL18A1
SETD7
SLC7A5
TXNDC5
TAM
CAPN2
MFRP
ANGPTL6
CCDC8

PLA2G10
CASQ1
PPP1R15B
UBASH3B
ABCC11
DENR
CAV3
TP63
IDDM17
TNFRSF6B
CES2
ARHGEF7
SQSTM1
WASF1
WNT3A
F2RL3
CD3E
LPAR2
SLC33A1
GGTLC1
MSC
DNER
CD14
GPR55
S1PR2
CD19
MS4A1
KLF4
CD163

GLP2R
COX5A
CD80
AIM2
TBPL1
NPEPPS
BCAR1
CD69
HDAC4
A1BG
HOTAIR
SOD2-OT1
HDAC6
KIF28P
BCL2L11
IBD20
INSL5
H3C9P
C4B_2
CDH5
TMED7-TICAM2
AAA1
CHAF1A
PARP2
MAMLD1
ARAP1-AS2
OCLN
VIM-AS1
GJC1

RBM14-RBM4
KLRC4-KLRK1
ABCC5
ERVK-20
DNM3OS
IL18BP
PTPRU
ARAP1-AS1
CDH11
OPN1MW3
HIPK3
CDH13
RASGRP1
LRPPRC
MBNL2
WASF2
CDK2
LINC01150
EMSLR
TRPM2-AS
ADAM10
NUTF2
DDX39A
GDF11
MRPS31
RABEPK
ABCC4
CDK2AP2
CDKN1B

LOC102724334
CBSL
STUB1
SIGMAR1
TP53COR1
LANCL1
TNIP1
LAMC3
CDKN3
TFG
MICU1
CITED2
CORO2B
PEMT
CPQ
RAPGEF3
CD52
RBM14
LYPLA1
C1D
VAV3
TOMM40
TIMM44
CAP1
CARM1
CEBPA
SEMA4D
FBLN5
CIB2

DEAF1
UBD
THRA1/BTR
CERNA3
SLC35A1
CCT4
GNLY
CENPA
SLCO1B1
SH2B2
IVNS1ABP
POSTN
CST12P
CELF1
TNFSF13B
GIPC1
TRAF3IP2
DHS
NEK6
SEPTIN9
WASF3
PGRMC1
USP19
MRPS30
MALT1
MMP24
CPSF4
ADCY3
PAPOLA

SUB1
RALBP1
PRSS21
BTG3
PDIA5
ERP29
EBNA1BP2
METAP2
IMMT
ERVK-32
SLC27A2
KIF2C
LIAS
RAPGEF4
LOC110806263
ESM1
NUP42
RPP14
PTPRT
FAF1
SLC7A9
LOC111674464
CHIT1
SLC2A6
MAP4K5
IRAK3
AKAP13
PTGDR2
DUSP12

IL17F
KLF12
LINC02605
SLCO2B1
RBPJL
SLC46A1
STK38
PHB2
TUSC2
AZIN2
TMEM54
LOC113664106
CHRNA4
ADCY8
TXNRD3
CYGB
MYSM1
C1QTNF7
MARCHF3
GPR146
CISH
H3P8
H3P28
H3P42
SLC5A11
CKM
RMI2
ERCC8
ABHD15

SNAP47
LRG1
ARAP1
TADA1
THEM4
DCD
RLN3
MSS51
CLCN7
CLK2
SERPINA3
CYP2R1
CLPS
PLIN2
CCR1
DEGS2
ACKR2
FOPNL
LTB4R
MSI2
OSCAR
CNP
CNR2
TSACC
COL6A3
WDR36
TAAR1
CPB2
CPD

KLF14
CLDN4
CPT2
CR2
CREB1
ATF2
CREM
FUNDC1
UPRT
CRY1
HT
SIRPA
CS
CACUL1
CSF1
CSF1R
PIWIL4
CSF2RA
CSH1
CSH2
CSK
SERPINA12
WIPF2
CILP2
ZNF569
CTAA1
CTBS
CTF1
PDIK1L

CTNS
SIK1
CTRB1
CTSG
ERFE
CTSK
RMDN2
UPP2
KLB
PPM1K
CYC1
SLC2A12
CYP1A2
AMOT
CYP2D7
LINC00599
CYP3A5
CYP4A11
CYP24A1
CYP27A1
DAB1
DGKQ
DAPK1
DAPK3
SPRED1
DBH
MARCHF10
DCX
TLE5

DEFA1
DEFA3
SGMS2
DES
NLRP6
PTCRA
DIAPH1
AFM
DIH1
DIO3
DLAT
DLX3
AGA
SARDH
DMRT1
DNAH8
DNASE1
DNM2
DNMT3A
DOCK3
DSC3
RCAN1
DSG3
HBEGF
DUSP4
DUSP9
TOR1A
E2F1
EBF1

EDA
S1PR1
AIC
EEF2
EFNB2
EGF
EIF2S1
ARID2
PAOX
EIF4E
EIF4EBP1
EIF4EBP2
AIF1
SERPINB1
ELAVL2
A2M
ENG
ENPEP
PRSS55
EP300
NRK
ERCC1
ERG
ESD
ESRRA
ALAS1
ETS1
EXT2
F5

F9
ALDH1A1
FABP3
FABP5
FBLN1
FAT1
LRRC55
FOLH1B
FBN2
EFEMP1
FBP1
RANP1
PHACTR1
FCGRT
HOXA11-AS
GPC4
FGB
ABCD2
FGF13
FGFR1
FGL1
FHL1
FHL2
MRAS
PUF60
VASH1
AAK1
ANKRD26
NLRP1

INPP5F
CARD8
SACM1L
KLRK1
MSRB2
TPX2
PDCD11
UNC13A
MYO16
NFASC
KDM6B
PASK
ATG4B
TBC1D1
NBEAL2
FBXO28
PLCB1
PHLPP1
FLT4
MCF2L
CUX2
ZNF629
SIRT4
ABCB10
FOLH1
NPTXR
TRAM1
BRD4
FOLR1

FOLR2
NNT
FOSB
RBFOX2
ELP5
SH3BP1
RAB38
TMEM245
PANX1
ZNF318
ALPI
FSHMD1A
ALPP
NR5A1
FUT2
FNDC5
ANGPTL5
SLC37A4
CNIH2
TCERG1L
NEGR1
GCA
TAFA5
ARIH1
TPSG1
BACE2
PART1
GALNS
GALNT3

TPGS2
KANK2
TIPARP
CHMP2B
SNED1
GORASP2
TKFC
ACOT11
LRIT1
GAS1
GAS6
PHGDH
FBXO25
FBXO8
ANKRD2
GBE1
GAPDHS
OPN1MW
GDF2
GREM1
GDF10
HS6ST3
GDNF
GEM
GFPT1
GHRH
GHSR
ABL2
GJA4

GJA5
ATP2C1
LAT
SND1
FOXP1
TAF5L
CACYBP
NAAA
GLS2
GLA
HCAR1
GPR162
PDCD4
RPS6KA6
GLI2
MAT2B
TOR2A
GLUD2
GNAT1
AMY2A
GP1BA
SLCO1B3
ANG
NEAT1
CXCR3
HECTD4
KSR2
CHAMP1
UTS2R

ZNF763
RSPO1
MCHR1
IGHD1-7
DLL1
TRBV7-8
GPR39
FFAR2
GPX3
GPX4
GRIA2
REM1
DEXI
LAMTOR2
OSTM1
FLVCR1
DBNL
GRIN1
GRIN2A
GRIN2B
STXBP6
CNIH4
DROSHA
GRM2
GRM5
CXCL1
GSN
GSTA4
GTF2H4

TRPM5
ICOS
GPSM2
EEF2K
NME7
SEC61A1
GPR132
NENF
DSE
CERS2
NRBP1
GYS1
SH3KBP1
H1-2
HABP2
HARS1
HBG2
HCRTR1
ANXA5
ERVW-1
NT5C
PIK3R4
UBE2K
HLA-DMA
HLA-DMB
HMGCS2
NR4A1
HNF4G
ONECUT1

SLC29A2
BPIFA4P
HNRNPK
HOXA3
HOXC4
APC
APCS
HPX
MMAB
HRH1
HES1
HSD3B1
HSD11B2
HSPA2
HSP90AB1
HSPG2
NDST1
HTC2
HTR2A
HTR2C
TNC
IBD2
IBSP
TMEM119
BRINP3
ARMH1
ID1
GADL1
IDDM3

IDDM4
TSPAN33
VWA2
IDDM11
NANOS3
CYCSP51
IFNAR2
SOX2-OT
IGF2R
IGFBP5
APOH
GPR142
ZACN
SLCO4C1
TICAM2
IL1R1
IL4R
IL6ST
IL7
IL7R
IL9
IL12B
ACADSB
IL15RA
FOXK2
ILF3
ILK
IMPDH1
ING1

ING2
AQP7
INSL3
INSM1
IRF1
AQP9
IRF7
ISL1
ITGAX
ITGB7
ITIH1
IVD
JAK1
JUNB
AREG
KCNC2
KCNE1
KCNH2
KCNJ3
KCNJ5
KCNJ6
KCNJ9
KCNN4
LINC-PINT
KIR2DL3
KIR3DL1
KLKB1
KRT8
KRT18

TMEM189
TMEM189-UBE2V1
SERPINA13P
LINC01550
CCL4L1
LIN28B
KTN1
C1QL3
HES3
LAMP1
RPSA
LCN1
LCP1
LDHB
LGALS2
LGALS3BP
LGALS4
SMIM10L2A
LOXL2
LRPAP1
GTF2H5
LTBR
MXD1
SMAD1
ARRB2
SMAD7
ASIC1
STS
MAP2

MBNL1
MBP
MC2R
SMCP
ADAM11
DNAJB9
MDH2
MDM4
MEOX2
MFGE8
MGAT2
KITLG
CXCL9
MITF
MLH1
MAP3K9
MAP3K10
MLN
FOXO4
MMP10
MNAT1
MPG
MPI
MPST
MPZ
MRE11
ABCC1
MS
RNASEK

CDNF
MSH3
MT1E
MT1JP
PLF
MTM1
MTTP
MUC1
MUSK
MMUT
MUTYH
MYF6
SERPINC1
MYL2
PPP1R12A
NAB2
NAGLU
NUBP1
NBN
NCAM1
NCK1
NCL
NDN
ATIC
NFATC3
NFATC4
NFIL3
ATP12A
NIDDM1

NINJ1
NNAT
NM
NME1
NOS1
NOTCH2
FXYD2
ATP2A1
NRTN
YBX1
NTS
NTSR1
NUMA1
TRIM72
OAS3
OAT
ATP4A
ODC1
ODF1
ORM1
CLDN11
OXA1L
OXTR
P2RX4
P2RX5
PA2G4
FURIN
PRDX1
MBL3P

PAM
PAPPA
PAX2
ASAP1
AK3
F11R
PCBD1
PCDH8
TMED7
TVP23B
PCMT1
TFB1M
METTL9
PCNA
RMDN1
INSIG2
GLRX5
MZB1
NT5C3A
CLEC1B
PDB1
PDC
DACT1
DCDC2
HSD17B7
PDE7A
SIRT7
RAB14
PDGFRA

GDE1
PIAS4
TRIM33
NBAS
ATP6V1H
SF3B6
RASD1
MPC1
ZFR
EMCN
MAP3K20
PER1
PF4
PF4V1
SERPINB6
PIM1
PKLR
PKM
PKNOX1
PLAGL1
PLAUR
PLCB3
IL17D
HDL3
PLEK
SERPINF2
PLK1
PLN
PLXNA1

PNOC
IL20RA
SLCO1C1
ACP5
FAM3B
H2BS1
PODXL
TREM1
SIAE
YIPF1
MOV10L1
POU1F1
SMOX
POU2F1
APBB1IP
POU2F2
ROBO4
DDIT4
EPB41L4B
POU4F1
ARL15
FEV
FBLIM1
CNNM2
DYM
VPS13C
NCAPG2
ACP3
TUG1

MARCHF1
MOCOS
PPP1R3A
MTPAP
MSTO1
RMDN3
AVPR1A
PTPA
RHOT1
FBXW7
AVPR1B
GIMAP5
HEMGN
PPY
AVPR2
PREP
PLXNA3
MIOX
RNPC3
ITLN1
FERMT1
SYBU
ENAH
PRKCD
USE1
ACOT13
GSDMB
GPRC5C
ZC4H2

LANCL2
NSFL1C
MYDGF
MAP2K3
MAP2K6
PCDHGA4
ZNF253
MASP1
PRSS8
CYP26B1
ASAH2
PSEN1
PSEN2
CLDND1
SUCNR1
SPHK2
NMUR2
ENY2
CHPT1
ADAMTS9
BAAT
AKR1B10
COQ9
PCBP4
KMT5AP1
GOPC
PSMD7
PSMD10
PELI1

ADAMTSL3
BAD
PTBP1
PTCH1
PTGDS
GJD2
PTGIS
HNP1
AS3MT
PTH1R
AHRR
SEMA6A
CFAP97
RNF213
MARK4
ZNF410
PTPRD
NEUROD4
NECTIN1
PCTP
OPRPN
ARHGAP22
RAC2
RAG2
RAN
RAPSN
GER
SCPEP1
NPS

RBP3
CCND1
RCN2
OPN1LW
REG1B
RELA
REST
RFC2
RGS1
RHD
RHEB
RHO
RIT2
RNASE2
SLC25A19
BRD2
ELOVL5
NIF3L1
GAS5
PROK2
RNY1
RNY3
ROBO1
ROCK1
HCN2
RP9
BCR
RPL10
RPL29

RPL36A
DELYQ11
RPS6KA1
HCC
RPS19
RREB1
RXRG
RYR3
S100A10
ACSM3
SATB1
CLEC11A
SCN2A
SCN7A
SCN10A
CCL16
CCL22
BID
CXCL11
SDC4
THADA
UBE2O
PCIF1
DMRTA1
SELPLG
SEMA3F
TSPYL2
LHPP
TNMD

ECSCR
STRA6
ABCG5
SRSF5
SRSF6
FBRS
IKZF5
SFTPB
SFTPC
SGCA
SMIM10L2B
GORASP1
PDIA2
WDR13
MFSD1
ST6GAL1
GNPNAT1
PMEL
AGXT2
SIX1
SKP2
SLC1A3
RTN4R
WNK1
SLC3A1
UBE2Z
SLC5A5
SFTPA1
SLC7A2

SLC9A1
SLC12A3
SLC14A1
SLC19A1
SLC20A1
SLC20A2
SMARCA4
CYP4F12
SMO
SNRNP70
H3P37
SOD3
BNIP3L
SORD
SORL1
SOS1
SOX4
SOX9
DST
SPINT1
SPR
SPRR2A
AKR1D1
SRY
ITPRID2
ST13
ST14
STAT2
STAT4

STC1
ELOVL4
STIM1
STX1A
SUV39H1
VAMP2
SYT1
SYT5
ADAM17
TAGLN
KLF5
TALDO1
TAP1
TAP2
TBX1
TBXA2R
TCF4
TCF19
TRD
BUB1
TDGF1P3
TDO2
TERF1
TGFA
TGFB2
TGFB3
TGFBI
TGFBR1
TGFBR2

THBD
THBS4
THRA
THRB
KLF10
TIE1
C1QBP
TJP1
TLE1
TLE3
TLR5
TNFAIP3
TPM4
CRISP2
C4A
C4B
TRPC3
INS-IGF2
TSC1
TSHR
TST
TTC4
C5AR1
POTEF
OPN1MW2
TNFSF4
UBE2V1
UCN
UGCG

UPP1
UPK2
USF1
KDM6A
VASP
VEGFB
VHL
EZR
VIP
VPREB1
LAT2
NSD2
WNT2
WNT5A
WNT6
WNT7A
XBP1P1
XK
XPC
XRCC1
YES1
SF1
CACNA1A
CACNA1E
ZNF236
DDR1
PSS
PXDN
TUBA1A

PAX8
FZD5
SCG2
SLMAP
SLC25A20
ASPSCR1
MBOAT7
FSD1
DDX39B
CALB1
PAGR1
NTT
NAA16
NLRX1
FZD3
SHCBP1
VASH2
CALM1
BICC1
ASRGL1
MOGAT2
STAM
SCUBE1
CDK5RAP3
FLAD1
PDGFD
COASY
CALM2
ECB2

CALM3
ZNF436
AAAS
KCNH6
HBHR
SLC38A1
TSC22D4
MAP1LC3B
AKAP1
MED25
CAPN3
UBL4A
TKTL1
CAPS
CAST
AXIN1
BRAP
CARS1
ELOVL3
SESN2
CRISPLD2
LOH19CR1
KCNK16
SETDB2
FSD1L
MIXL1
MAGT1
TAGLN2
TKTL2

SLC41A2
EEA1
ASCC2
NLRC5
MEHMO
SYVN1
HOPX
CUL1
ZGPAT
KISS1R
SPZ1
IRS4
ATP5MD
HAVCR2
ORAI1
PLXDC2
ELP1
CST7
KRT90P
LGR5
MAPKAPK5
GALP
MADD
STC2
ABCB11
NCOA1
DYNLL1
VAMP4
CBLB

DGAT1
HRK
TNFSF14
CBS
FADD
SIGLEC5
TNFRSF11A
TNFRSF10B
SUCLA2
TRIM24
DPM1
NRP1
CCN5
CCN4
ASAP2
CCKBR
SPHK1
FUBP1
EIF2S2
TRPA1
UCN2
CH25H
KNSTRN
BMF
RPL14
GPRC5A
SLC7A7
PYGO2
MTA1

CD1D
SLC16A4
SLC16A3
ARHGEF1
PDCD5
HGS
PCSK7
ARHGEF2
RFT1
TIMD4
ARRDC4
HACD1
CTBP1-DT
MTG1
CD5L
PTTG1
NAF1
NOG
CD8A
MFHAS1
STK17B
PIWIL1
SLC38A5
ACVR2B
B4GALT5
SLIT2
LONP1
CD27
CIAO1

CD101
CD28
CYP7B1
TP53INP1
NCR1
MAP4K4
CD33
HOMER1
FHL5
PCYT1B
CHST3
ROCK2
NAPSA
MAPK8IP1
SCARB1
ADAMTS4
ADAMTS1
GAL3ST1
BAG3
EI24
CIR1
CCL4L2
PRDX6
CD47
IKBKE
SOCS5
LPIN2
SEMA3E
SART3

HDAC9
ADGRE5
RASSF2
PIEZO1
IP6K1
GIT2
TBC1D4
NUAK1
RNF10
MVP
TNFSF15
CCS
CDC42
CDH1
CASP8AP2
HMG20A
GPD2
UBE2E2
AP3S2
MAEA
GRB14
PEPD
CMIP
ITGA1
PLEKHA1
ZFAND3
DGKD
CCND2
PSMD6

NFATC2
ZNF257
CNKSR2
JADE2
AUTS2
EPC2
GP2
IL13RA1
RNF6
SCTR
SLC1A2
BRAF
FAM234A
USP48
VPS26A
SFRP4
SLC22A3
HK1
ATP2A3
HLA-DRB5
CCDC92
CDO1
NUS1
TMEM155
KCNU1
SMAD5
MAT1A
NKX6-1
ZC3HC1

BCL2L1
S100A6
BHMT
TRPC1
NFE2L1
UGT1A6
UGT1A1
PRKCE
MAPK9
NR1D1
CASP12
FEM1B
ACOT2
ABCC2
COL3A1
CRHR1
AANAT
CYP11A1
DAG1
ARX
ANKRD23
AK1
EPAS1
ALDOB
NCOA6
SDF2L1
HSD17B3
KCNMB1
PHOX2A

MKI67
MTR
OXCT1
ATP5F1B
PPP2CA
PRKCI
SCN1B
SRC
STAR
CASP7
MAP1LC3A
GPT2
EIF3F
GADD45GIP1
ANK1
GRK5
KIF11
TM6SF2
AGMO
MAP2K7
TFAP2B
TMEM163
DGKB
COBLL1
PRPF31
LAMA1
ALDH7A1
POU5F1
AHI1

ZMIZ1
RBMS1
ZRANB3
PRC1
MPHOSPH9
TRDN
CAMKK2
TSBP1
EHMT2
SPPL3
POC5
NDUFAF6
CTBP1
LINGO2
SLC16A13
ADGRL3
MACF1
ALK
ABCA4
TTLL6
RBPJ
SNHG17
MCM6
TRPM1
NRL
OAS1
GATAD2A
POMGNT1
BCL9

ROM1
ATXN1
SGCD
SGCG
SSR1
TUB
CLIP2
DHDDS
PBX4
CAMK2G
STX8
NR2E3
SUMO2P17
HMGA2-AS1
LINC00271
LMCD1-AS1
ENPP7P10
OSER1-DT
PROX1-AS1
UBE2E2-AS1
LINC01426
LINC00844
ADAMTS9-AS2
PRC1-AS1
ARPIN-AP3S2
SCAANT1
TSPAN3
FRY
HNF4A-AS1

LINC00824
LINC02010
PKN2-AS1
C5orf67
TSBP1-AS1
LINC01339
PABPC4-AS1
ETS1-AS1
HORMAD2-AS1
TOPORS
CALCOCO2
RAMP2
LINC02576
DLEU1
CCND2-AS1
ADARB1
MERTK
LINC02841
LINC02484
LINC02030
LINC01611
PRPF8
YKT6
RAI1
PNPLA6
TRIOBP
POLR3A
NUDT6
MGLL

CHUK
OSBPL1A
OSBPL7
TTC8
CYB5D2
TTC39C
CNGB1
CNGA1
EDARADD
CHMP4B
ZNF513
ARL9
SLC9B2
SDHAF4
UNC5D
HGSNAT
MAGEC3
CRX
ZSWIM3
RFLNA
BEST3
RDH12
RTN4RL1
HORMAD2
LINC01010
C8orf37
DARS1
TTLL8
RPSAP52

CLN8
EYA2
DLEU7
FGF14
CPEB3
NLGN1
RALY
RAB3GAP1
SNRNP200
ZHX3
ZZEF1
CEP68
FBXL7
RPGRIP1L
ARHGEF18
CRB1
ABCB9
ARL2BP
AMACR
GABRA4
ST6GALNAC3
GABRG3
FSCN2
SAMM50
ABI3BP
PTPN23
OSBPL3
GPSM1
IFT172

VPS33B
TBL2
NSG1
PALD1
HNF1A-AS1
C17orf58
KIF9-AS1
PPIL6
ZNF775
GTF2I
GUCA1B
PURG
LRP12
FSCN3
HIVEP2
HLA-DOB
HLA-DRB9
IDH3A
IDH3B
EYS
CERKL
KCNQ3
PCARE
CFAP77
LIMK1
LINC01344
IL17REL
LINC01122
LINC02245

ARL3
C16orf74
MAK
PIM3
MCC
ASCL2
MOG
ND4
TRNH
NEK2
NFKBIL1
NTRK2
NTRK3
SCAPER
PCSK6
IMPG2
ASB3
HSD17B12
SNX7
PDE6A
ANKFY1
PDE6G
PEX5L
PDE6B
PLCD1
LRP1B
PELO
PRRX1
EXOC6

GIN1
CNTLN
TXNL4B
PINX1
SRBD1
DARS2
SBNO1
PPP2R2C
PRIM2
INTS8
MINDY1
SPATA7
UBAP2
ARHGAP15
KIZ
KLHL7
TEX14
ANKH
RELN
C1GALT1
PDSS2
ATP8B2
PTGFRN
LRFN2
GPR158
STARD9
PITPNM2
UVSSA
KIAA1549

SLC7A14
NECTIN2
SINHCAF
SENP2
PRPH2
RFC1
RGR
RGS7
RGS13
RLBP1
MRPS35
AGBL5
RP1
RP2
RPGR
RPE65
MRPL12
SAG
ATXN2
ATXN7
PIEZO2
BLM
XYLT1
KIF9
SEMA4A
AKTIP
YTHDC2
ST3GAL3
MLX

TGFBR3
TNFAIP6
TULP1
ZBED3-AS1
USH2A
CLRN1
VSNL1
ZIC1
ZSCAN20
CA4
PRCD
GDAP1L1
GCC1
ZNF408
THSD4
SCD5
CPED1
PCNX2
SPHKAP
TNKS2
ZNF34
IFT88
NPRL3
LRMDA
CTTNBP2
FAM161A
CHCHD6
OFD1
OASL

PABPC4
MTMR3
CCDC149
PRPF4
PRPF3
CCNQ
CDHR1
LRAT
REEP6
NRXN3
NRXN1
ZNF101
MED23
GTF2IRD1
CELSR1
WSCD2
DEPDC5
UBE3C
DOCK4
IFT140
DHX38
MTSS1
SFI1
MAGI2
FCHSD2
RHOBTB1
RBM19
USP3
BCL11A

SLC2A11
CRY2
TMBIM4
SAT1
STAM2
FANCD2
IL37
PYCARD
HDLBP
IL5
DPPA3
KCNJ4
HEBP1
AZU1
SULT1E1
HIRA
SLBP
LPAL2
XPR1
LIPG
ARHGEF11
RNU1-1
GPD1
RAPGEF1
C1QTNF9
NPC1
TAS2R13
PDE3B
METRN

SLC39A7
PTGES2
SNAP23
EBI3
CDK9
SPRY2
C1QTNF1
CMA1
CYP2C18
PHOSPHO1
TIMM8A
DRD2
TMEM154
ENSA
FCGR3A
FCGR3B
ERP44
SIRT5
CERS6
POU2F3
C5AR2
METRNL
GRB10
HCCS
MCIDAS
APOD
ITGA2
ITGA2B
KCNJ15

RNR2
OSM
TNFRSF12A
PGR
PARL
CENPJ
PRKG1
PRTN3
CCNL1
ALX4
RRAD
RXRA
CACNA1D
GGCT
ELOVL6
STEAP4
SP6
WNT5B
IL18R1
LITAF
P2RX5-TAX1BP3
LINC-ROR
CDKN1A
CIB1
ATG7
TXNRD2
PDLIM5
LARP1BP2
RNU6-392P

CES1
ADAM28
SLC27A4
NISCH
FITM2
COX4I1
GNPDA2
R3HDML
CYP2C8
DHCR7
ARID3A
EPHX2
FABP6
FCGR2B
KIF6
GPC5
P2RX2
GGA3
ICOSLG
ARHGEF12
LARS2
NPAS4
SLC39A1
SPINK4
GPS2
RMC1
KCNIP1
HTN1
IKBKB

ACAT1
LDHA
LECT2
LMX1A
CIMT
DNAJB3
DEFB103A
MMP7
MMP12
MMP13
MRC1
MT1X
MTHFD1
ACLY
NF2
OCA2
OPRM1
P2RX1
P2RY2
DUOX2
PAK1
BFAR
HDAC7
GPRC5B
PEG3
PFKM
PIK3C2G
PLD1
DUOX1

NANS
TERF2IP
TMEM132A
CEP55
QRSL1
RNLS
SRGN
PRKAB2
SLC30A6
ATF7IP
CNDP2
DOK5
SULF2
AZGP1
MEPE
ABHD6
ARID1B
KLHL42
PREX1
MAGEE1
NCOA5
PTPN9
PVALB
FAM3A
RRAS
TSPAN31
MSMO1
SDC1
SDC2

XYLT2
SLAMF1
SLC16A1
TF
SERPING1
TRH
UGT2B15
VIPR1
VLDLR
VTN
SLC30A1
GHS
ANKRD55
ACAD10
CAMP
ARID5B
FCN3
KLF7
SOCS2
TSC22D1
PER2
P2RX6
IL32
SFXN1
GFPT2
CDH2
AKT3
OCTN3
MICC

HMGA1P8
DIP
WASHC1
LINC00994
GNG12-AS1
DEFB4B
BOP
ALL2
LY75-CD302
RPS10-NUDT3
COMMD3-BMI1
PLUT
PREB
RBM6
MPHOSPH10
CDK6
FLOT1
OLIG2
THRIL
ZMPSTE24
FSTL3
LOC102723971
LOC102723996
TCIRG1
WARS2
DLC1
ATP8A1
SPON2
SPON1

IFI30
FAM3C
CDX2
SALRNA1
UNC13B
TEMPS
PROCR
AGPAT1
SPTLC1
OLFM4
LRP1-AS
NPC2
USP16
IGF2BP1
IGF2BP3
SIRT1-AS
CTCF
CD226
MCS+9.7
EBP
CAPN9
LOC107832851
SLC17A3
STARD10
CGA
CPLX1
CGB3
CEACAM3
PPP1R13L

CCL27
CYP46A1
CEACAM7
CD300C
NMU
FGL2
EHD1
CHEK2
CHM
CHN2
BIRC8
MGAT4A
CHRNB4
AD7
TIRAP
OSBPL8
CIDEA
CKB
OLIG1
AP3S1
CLCN2
CLCN3
CLCN5
GGTA2P
SPIC
TPPP2
TCHHL1
LYPLAL1
KLHDC7A

COL2A1
PIFO
COL4A2
COL4A5
COL8A1
COL9A1
COL9A2
ACVR1C
COL17A1
FAM3D
MAP3K8
COX5B
COX7A1
COX10
MTPN
LRGUK
CPT1B
GPAT4
CRABP2
ADORA3
ROMO1
MT1P3
FGFBP3
SESN3
OR2AG1
CSHL1
GSC
BEAN1
CD300LG

CST2
PTPRVP
SLC30A7
IL23R
FAM78B
CLDN19
CTRL
CTSH
CYB5A
CYP1B1
CYP2A6
BRINP1
DBI
AKR1C2
DDX3X
DQX1
DEFB4A
CFD
AAVS1
DIAPH2
SEPTIN1
DMBT1
DMP1
DNTT
DRD3
DUSP6
EBM
TYMP
LPAR1

EFNB3
EGR2
EGR3
MLKL
EIF4A2
EIF4G1
ELAVL1
TET3
MARK2
CENPX
EPHA4
EPHX1
ESRRB
ESRRG
ETFA
EVC
EXT1
F2RL2
MED19
UNC5B
SKA1
MARCHF8
ALDH3B1
JMJD1C
SLC39A12
FCP1
SLC29A4
FDXR
FGF11

FHIT
COG2
MLXIP
SBNO2
RAB18
NT5C2
FAIM2
KDM1A
CAND2
TBC1D9
SYT11
SIK2
ARC
COBL
TTC28
SYNE1
PSD3
CRTC1
DICER1
NCS1
ZFPM2
MLYCD
TRS-AGA2-3
SEC14L2
FAM215A
ZMYND8
LY96
SPESP1
ALPL

FUT6
HCG27
CADM2
AKNAD1
SLC41A1
CASC2
ANKK1
GABPB1
GABRG2
SLC39A6
KLK5
MGAT4C
GALNT2
SGMS1
TENM4
ATRNL1
KIFBP
PITPNC1
KLHL3
MYCBP
GBP2
HSPB8
NUPR1
CNNM1
GDF1
PCOLCE2
TAS2R12P
RNU12
GH2

GJA8
ACAD8
DKK4
DISC1
GLB1
SERP1
SIT1
SESN1
DHDH
PDLIM3
EIF3K
MCAT
BIN1
PCLO
AMY1A
AMY1B
AMY1C
GOLGB1
SFN
GPI
GPM6A
GPR1
AQP11
H19
MUC19
LINC00523
PGP
HCCAT5
CISD3

KCTD1
GPR21
MIF-AS1
TTC28-AS1
IGHD1-14
SLC9C1
CYP4V2
LY86-AS1
FFAR3
ANK2
GRB2
SLC27A6
RGCC
MCTS1
KLF15
MYLIP
CTNNA3
GSTA1
GTF3A
HOOK2
GYG1
RHOD
SLC39A3
SLC2A8
ACAA1
ERO1A
TBX21
SLC40A1
HSD17B10

HADHA
HADH
HAS3
HBA2
CFHR2
HLA-DOA
HLA-DRA
HLF
HMBS
FOXA3
HNRNPA1
HNRNPF
HOXA5
HRAS
BIRC2
BIRC3
HTR2B
HTR4
ICAM3
ZFP69
APOC2
HSD17B13
ACTBL2
APOC4
LINC01193
IGFBP4
IGH
IK
FASLG

AQP2
IL12A
IL16
IMPA1
AQP5
INSIG1
KCNB1
KCNC4
KCND3
SLC27A1
KCNJ10
KCNK3
KCNN2
KCNN3
KEL
KIR2DS1
KIR3DL2
KPNA1
KRT7
CHKB-CPT1B
RHOA
KRT19
KRT31
LEKR1
L1CAM
RND3
LAMB3
STMN1
LIPA

FADS3
SAMD12
TOMM5
LPP
CYP4F3
LTF
LY9
LY75
SH2D1A
TNFSF12-TNFSF13
MAN1A1
MAN2A1
MAX
ARSL
MC5R
ME1
MEF2C
MGST3
KMT2A
ALDH6A1
EIF2AK4
PLIN5
POTEKP
PGBP
MSH2
MSH5
MT1B
MT1F
MT1G

MT1H
MT1M
MT1L
ATP8
RNR1
MTRR
MYF5
MYH6
MYLK
MYO5A
ATHS
NFYA
NFYB
NFYC
NPM1
NPR1
NPR2
NPR3
NPY2R
NTRK1
NUCB1
NR4A2
OMG
G0S2
SERPINB2
SLC35B3
IRAK4
PCOLCE
IPO11

IGF2-AS
DNAJC27
BPIFA1
WAC
CSAD
HACD3
LARS1
SCLY
PDGFB
CYRIB
PDGFRB
LSR
ATP5MC1
PDHB
PDK2
WWOX
PFDN2
PFKFB2
PFKFB3
SERPINE2
PIK3C2A
PIK3C3
PLA2G4A
SHC3
PMAIP1
PML
ATP7A
FXYD5
ATP5PO

PNLIP
SEPTIN5
SLC38A2
NLGN3
ZFAND6
EXOSC4
POU3F1
UGT1A10
UGT1A8
UGT1A7
UGT1A5
UGT1A9
UGT1A4
UGT1A3
SLC6A20
TRPM4
BNC2
PPP1R2
ENOX1
APPL2
NAT10
NEIL3
LARP6
LIN7C
LGR4
CAMK2N1
SAGE1
VPS35
UGGT2

PRKD1
DEFB103B
DMAP1
NDUFA12
SLC50A1
ALG1
EIF2AK2
METTL3
CCL28
TMPRSS15
MMP26
PSG2
CELF4
PSMA7
SMARCAD1
CA10
ARNTL2
PSMB6
SLC12A9
HYMAI
PSMD2
PSMD3
CPA6
SLC39A10
ATP10A
PTAFR
PTGER3
MRS2
KIDINS220

XPO5
PTMA
USP36
PTMAP4
TSHZ3
CHD8
USP37
MIER1
SPTBN4
PTPN4
CCAR2
PTPN11
PTPRB
PTPRS
CXCL16
PYGB
BCAT1
RAB4A
RAB5B
RAB27A
RAG1
RANGAP1
RARB
REL
REV3L
EXOC4
RNH1
RORB
OPN1SW

RPE
BCS1L
RPN2
RPS6KA2
RPS6KA3
CLIP1
RTN1
S100A4
SAA1
SAA2
SCN8A
SCO1
CCL3
CCL7
CCL8
XCL1
CX3CL1
SDHA
SLC17A9
POTEM
POU5F1P3
NSD1
NFKBIZ
GMCL1
GMCL2
BLVRA
DCLRE1C
MT1IP
CHCHD2P9

POU5F1P4
BMI1
FNDC4
RFX7
SLC30A5
SIX3
SKIL
ACD
SLC1A5
SLC1A7
FRTS1
WNK4
WASH6P
SLC8A1
SLCO1A2
SLPI
BRD9
GRAMD2B
SIGLEC1
BNIP3
SOX5
BPI
SRI
SSTR2
STX5
SYT4
TAC3
TACR3
TADA2A

TAF1
TAL1
TFAP2A
TIAM1
TLL1
ACTG1
NR2E1
TOP1
TOP2A
TPM1
HSP90B1
TRAF3
ACTG2
CAPN5
C5
TYROBP
UBE2B
WARS1
XDH
YWHAG
CA5A
CACNA1C
CACNB3
DUSP26
VKORC1
KCTD15
LY6G6E
PRRC2A
IRX3

LAP
TFEB
HBFQTL2
TNFAIP8L2
NEIL1
BBS10
MAFK
PRR5L
ADM2
STN1
FOSL1
TSEN2
COLEC12
CAMK2A
MOB2
NIPA2
SLC14A2
TLR10
ZNF239
PICALM
INHBE
GPR61
IMMP2L
HORMAD1
KAT8
PPP1R1B
PHF6
C7orf50
ZBED3

DYRK3
L3MBTL3
DOC2B
ZNF469
GLYR1
MFSD9
PTPN5
IGSF21
DGKZ
API5
SLC22A16
PIAS1
CAV2
RUNX1
JRK
HSD17B6
RUNX3
VAMP8
PAGE1
MBTPS1
ADAM19
CBR1
CBR3
TNFSF12
RAB11A
RIPK2
DLK1
GALR2
BANF1

TMEM11
EIF2B5
CCND3
NOL3
KALRN
TAF1C
EXOC3L2
CCRL2
PAPSS2
USP14
USP2
MTMR7
TRIM47
HTR3B
ZBED1
CD247
SLC16A7
CD4
DSEL
LARGE1
GORAB
ITGB1BP1
ADGRG1
PRDM6
CGB5
TGFBRAP1
TMPRSS11D
PEX16
CGB8

TJP2
OPN4
ITM2B
AKAP6
ATG5
ADAMTS2
STXBP5L
CXCL14
ENTPD3
GDF3
KIF20B
WTAP
NCOR1
NCOR2
CD48
EIF5B
AQR
ECE2
TOX
HEPH
SGSM2
MAFB
EXOG
THRAP3
KCNE2
SCO2
NODAL
SUFU
ERGIC1

CDON
OTX2
FGF8
NKX2-5
SOX3
VANGL1
ZIC2
HESX1
TRIM8
DISP1
FOXH1
TRIP11
TGIF1
ARNT2
LZTR1
FUZ
SLC29A1
HIF3A
PRLR
MFSD2A
HLA-G
DALIR
SLC26A6
LOC646736
ADK
COL6A2
TBL1X
GCFC2
SLC7A1

CLK1
TRG
PTBP2
APMAP
RAP1A
SDHC
IL34
HPSE2
PAPPA2
RPS14
CDK5R1
CCNG2
EBAG9
PROK1
IL1F10
RAMP3
PRG4
CALCRL
MS4A3
NFE2L3
SH3PXD2A
CDK1
TPH1
SDS
ADAMTS5
CDCA5
COL14A1
UPK3B
HKDC1

ANC
PAIP1
CEACAM8
VIPR2
INO80B
LCT
QPCT
UTS2B
C5orf34
GCHFR
LHCGR
PCBP1-AS1
LUM
LGALS13
HOXD13
GYS2
GZMM
CD209
B3GAT1
PITX3
VIT
PIKFYVE
ABHD5
ELF5
PLAC8
UBR5
NR0B1
POLR2G
DGCR8

LGALS14
ASCL1
MGAT1
MEST
FLOT2
PLXND1
FGR
TAS2R9
FCN2
BDKRB1
GPAM
ACOT1
S1PR5
YWHAH
ACOX1
CHRM2
AIFM1
S1PR4
NT5E
NPY1R
HCN3
WDR35
PSMB9
PPAT
PHKA1
ABCB4
UCHL5
PDE3A
PDYN

PFAS
ATP4B
ATP5F1A
OPRK1
OTC
PNMT
PYGM
CALD1
CASP6
SOAT2
KMT5C
PRKRA
UGT2B17
UCK2
UMPS
TOMM70
SLC28A2
RGN
ABCC3
GMPS
CCNA2
CCNB1
CCNT1
QDPR
SCP2
SSTR3
SLC28A3
RAP1B
RASGRF1

BCKDHA
BCKDHB
GRK1
BDH1
BDKRB2
TPI1
AACS
SSTR1
ERCC4
ABAT
DRD1
ACSL3
FGA
AOC1
GCNT1
GHRHR
GABBR1
SCAP
ACSL6
GJB6
TREX2
ADSS1
GJD3
ABCC9
ATP5PD
ACMSD
ADSL
CYP11B1
DHODH

KHK
ARG2
KCNA5
KCNA2
ITGA3
ITGB1
ITGB3
JAK3
NCF2
NEFM
NEFH
ASS1
LTBP1
GUK1
HDAC1
GSK3A
GJB2
CACNG4
HMGCL
HMGCS1
GNAT3
HCN1
HNRNPA2B1
HPRT1
SLC51B
SLC51A
PRKCSH
AGGF1
RDX

RGS2
WNT3
TYR
SMPDL3B
ETV5
CYLD
MFT2
MSN
GLOD4
SLC4A2
RELB
SLC4A8
AMBRA1
CAPNS1
MTMR9
INTS3
TPM3
FANCA
DACH1
LINC02210-CRHR1
PTRH1
SNU13
PTRH2
PDK1
PNPLA8
NOTCH4
C1QTNF6
PRKCQ
IL19

NDFIP1
CDR3
DOCK8
DDX27
AICDA
SLC3A2
FCGR1B
JMJD6
ABL1
GZMA
FCGR1A
SPRY1
ECI2
USP18
CCR7
LAG3
IDDM6
IDDM8
IFN1@
SRD5A2
TERT
ESR1
G6PD
FFAR1
NR3C2
MTNR1B
PTPN1
SHBG
VDR

GPBAR1
HMGCR
ACHE
FFAR4
AR
IDO1
CYP19A1
ACACB
SREBF2
PTPN2
RORC
CA2
GC
MAOA
NR1H4
NR1H3
FGF1
NPC1L1
MTNR1A
ABCB1
PTPRF
SLC10A2
CA1
SRD5A1
CD81
ESR2
FAAH
NR1H2
CYP17A1

GLS
PTPRC
SHH
ENPP2
SCGB1A1
TDGF1
TEP1
TERF2
TRIM9
UGT1A
