# Supplementary material for: Network pharmacology of bioactives from Sorghum bicolor with targets related to diabetes mellitus
Source: PLoS One. 2020 Dec 31;15(12):e0240873. doi: 10.1371/journal.pone.0240873 (PMC7774932; doi:10.1371/journal.pone.0240873)
Supplement: S3 Table — (PDF) [file pone.0240873.s003.pdf]

A list of the overlapping 118 genes between SEA and STP
AKR1B1
CDA
ADK
ADORA3
HSPA5
HSPA8
ADORA1
TOP1
ADORA2A
GAPDH
POLB
ADA
AHCY
TK1
GBA
PDCD4
EHMT1
CA9
CA12
CA14
CA1
POLA1
OGA
PNP
GRK1
CA2
EHMT2
PAM

LPAR6
CES2
MPEG1
DNM1
FAAH
PRKCA
EPHX2
ENPP2
ACP1
CDC25B
HAO1
CNR1
FABP3
CNR2
ASAH1
HSD17B3
TRPV1
HMGCR
S1PR3
PTPRC
SLC22A6
RARB
PPARG
TOP2A
LPAR5
PPARA
CDC25A
CA3
CA7

OXER1
PHF8
GSTK1
LTB4R
KDM2A
PTGER2
TBXAS1
TBXA2R
PTGER4
PTGIR
PLA2G4A
FFAR4
GABBR1
PTGER3
FABP4
PTGFR
PPARD
S1PR1
KDM5C
MGLL
PRKCE
ALOX5
FFAR1
ALOX12
STS
CYP17A1
NPC1L1
GPBAR1
SRD5A2

G6PD
SHBG
SERPINA6
CDC45
VDR
NR1H3
NR1H4
AR
ESR2
RORA
CYP19A1
SREBF2
ESR1
SHH
RORC
ABCB1
NR1H2
PHLPP1
AKT1
CA5B
CA6
CA5A
CA4
CA13
MAOB
NQO1
PGR
NFKB1
CYP1B1

MAOA
HNF4A
DRD4
