# Supplementary material for: Network pharmacology of bioactives from Sorghum bicolor with targets related to diabetes mellitus
Source: PLoS One. 2020 Dec 31;15(12):e0240873. doi: 10.1371/journal.pone.0240873 (PMC7774932; doi:10.1371/journal.pone.0240873)
Supplement: S4 Table — (PDF) [file pone.0240873.s004.pdf]

A list of 81genes related to 16 compounds

AKR1B1
CDA
ADK
ADORA3
HSPA5
ADORA1
TOP1
ADORA2A
GAPDH
ADA
PDCD4
EHMT1
CA1
OGA
GRK1
CA2
EHMT2
PAM
CES2
FAAH
PRKCA
EPHX2
ENPP2
ACP1
CNR1
FABP3
CNR2
HSD17B3

TRPV1
HMGCR
PTPRC
RARB
PPARG
TOP2A
PPARA
OXER1
GSTK1
LTB4R
TBXA2R
PLA2G4A
FFAR4
GABBR1
PTGER3
FABP4
PPARD
S1PR1
MGLL
PRKCE
ALOX5
FFAR1
ALOX12
STS
CYP17A1
NPC1L1
GPBAR1
SRD5A2
G6PD

SHBG
VDR
NR1H3
NR1H4
AR
ESR2
CYP19A1
SREBF2
ESR1
SHH
RORC
ABCB1
NR1H2
PHLPP1
AKT1
CA6
CA5A
CA4
NQO1
PGR
NFKB1
CYP1B1
MAOA
HNF4A
