# Supplementary material for: Network pharmacology of bioactives from Sorghum bicolor with targets related to diabetes mellitus
Source: PLoS One. 2020 Dec 31;15(12):e0240873. doi: 10.1371/journal.pone.0240873 (PMC7774932; doi:10.1371/journal.pone.0240873)
Supplement: S1 File — (DOCX) [file pone.0240873.s005.docx]

**Research highlights**

ᆞPPAR signaling pathway is a hub signaling pathway of SB against T2DM.

ᆞSIX genes and FOUR compounds are associated with PPAR signaling pathway.

ᆞFOUR compounds in SB may act as glucose homeostasis agents against T2DM.
